# How to limit the speed of a motor: the intricate regulation of the XPB ATPase and translocase in TFIIH

**DOI:** 10.1093/nar/gkaa911

**Published:** 2020-11-16

**Authors:** Jeannette Kappenberger, Wolfgang Koelmel, Elisabeth Schoenwetter, Tobias Scheuer, Julia Woerner, Jochen Kuper, Caroline Kisker

**Affiliations:** Rudolf Virchow Center for Integrative and Translational Bioimaging, Institute for Structural Biology, University of Würzburg, 97080 Würzburg, Germany; Rudolf Virchow Center for Integrative and Translational Bioimaging, Institute for Structural Biology, University of Würzburg, 97080 Würzburg, Germany; Rudolf Virchow Center for Integrative and Translational Bioimaging, Institute for Structural Biology, University of Würzburg, 97080 Würzburg, Germany; Rudolf Virchow Center for Integrative and Translational Bioimaging, Institute for Structural Biology, University of Würzburg, 97080 Würzburg, Germany; Rudolf Virchow Center for Integrative and Translational Bioimaging, Institute for Structural Biology, University of Würzburg, 97080 Würzburg, Germany; Rudolf Virchow Center for Integrative and Translational Bioimaging, Institute for Structural Biology, University of Würzburg, 97080 Würzburg, Germany; Rudolf Virchow Center for Integrative and Translational Bioimaging, Institute for Structural Biology, University of Würzburg, 97080 Würzburg, Germany

## Abstract

The superfamily 2 helicase XPB is an integral part of the general transcription factor TFIIH and assumes essential catalytic functions in transcription initiation and nucleotide excision repair. The ATPase activity of XPB is required in both processes. We investigated the interaction network that regulates XPB via the p52 and p8 subunits with functional mutagenesis based on our crystal structure of the p52/p8 complex and current cryo-EM structures. Importantly, we show that XPB’s ATPase can be activated either by DNA or by the interaction with the p52/p8 proteins. Intriguingly, we observe that the ATPase activation by p52/p8 is significantly weaker than the activation by DNA and when both p52/p8 and DNA are present, p52/p8 dominates the maximum activation. We therefore define p52/p8 as the master regulator of XPB acting as an activator and speed limiter at the same time. A correlative analysis of the ATPase and translocase activities of XPB shows that XPB only acts as a translocase within the context of complete core TFIIH and that XPA increases the processivity of the translocase complex without altering XPB’s ATPase activity. Our data define an intricate network that tightly controls the activity of XPB during transcription and nucleotide excision repair.

## INTRODUCTION

The ATPase activity of the superfamily 2 (SF2) helicase XPB is indispensable for two main cellular processes, transcription and nucleotide excision repair (NER) (1–4). XPB is part of the general transcription factor IIH (TFIIH) (5–7), which is functionally divided into core TFIIH and the CAK complex. Besides XPB, the core complex contains a second SF2 helicase, XPD, as well as the p62, p52, p44, p34 and p8 subunits. The CAK (cyclin-dependent-kinase (CDK)-activating kinase) complex completes the TFIIH architecture and consists of MAT1, Cyclin H and the kinase CDK7. Holo-TFIIH orchestrates the three differential enzymatic activities of XPB, XPD and CDK7. The individual roles of the three enzymes present in TFIIH differ depending on the cellular processes that TFIIH is involved in. During RNA polymerase II (RNAPII)-based transcription the ATPase activity of XPB is required for promotor opening, while XPD solely assumes a scaffolding function (6,8–11). Phosphorylation of the C-terminal domain (CTD) of the Rpb1 subunit of RNAPII by CDK7 within the CAK complex promotes the initiation and elongation in transcription (12–15), whereas the same complex assumes an inhibitory effect on the NER activity and was shown to dissociate from core TFIIH prior to the incision of the damaged strand (16–18). In contrast to transcription initiation, both the ATPase activity of XPB and the ATP-dependent helicase activity of XPD are essential for the NER pathway to anchor TFIIH to the site of the damaged DNA and to open the DNA duplex in the context of the lesion (6,8–11). The significance of an intact TFIIH complex for NER is reflected in its association with three severe autosomal recessive disorders. Mutations in the *ERCC3* (XPB) or *ERCC2* (XPD) genes can cause xeroderma pigmentosum (XP), trichothiodystrophy (TTD) and sometimes combined features of XP and Cockayne syndrome (CS) (19–22). No disease mutations in other core TFIIH subunits are known, except for mutations in the *GTF2H5* (p8; also referred to as TTD-A) gene, which are known to cause TTD (23).

So far, all patient mutations found in the *ERCC3* gene are located in domains other than its two RecA like domains (HD1 and HD2), which harbor all seven conserved helicase motifs (22). A crystal structure of an archaeal homolog of XPB from *Archaeoglobus fulgidus* revealed in addition to the helicase domains the presence of a damage recognition domain (DRD) and two unique motifs named the thumb like motif (ThM) and the RED motif (4). The human XPB protein is substantially longer than its archaeal homologue and contains N- and C-terminal extensions. In order to perform its ATPase activity, XPB is suggested to bind the DNA duplex by bringing its ThM and RED motifs into close proximity to each other (4) and mutations in either motif were shown to exhibit a reduced DNA-dependent ATPase activity comparable to a mutation in the Walker A motif (K346 in human XPB) (9). However, rather than being a ‘true’ helicase, XPB more likely functions as an ATP-dependent DNA-translocase and it was shown that its ATPase activity in transcription is utilized to track along the non-template strand in a 5′-3′ direction (24,25). Analogous to the stimulation of XPD’s helicase activity by p44, the enzymatic activity of XPB is also regulated by its binding partner p52 (6). The importance of the interaction between XPB and p52 is demonstrated by the severe effects of an XPB mutation (F99S) found in XP/CS patients. The F99S mutation was shown to weaken the interaction between XPB and p52, resulting in a highly reduced ATPase activity of XPB (6). However, so far, it is not known how this regulation is achieved. The lack of structural information further hindered an understanding of the functional interaction between XPB and p52. Recently, cryo-EM structures of the human and yeast TFIIH complex at resolutions between 3.5 and 4.7 Å (26–29) were solved and shed light on the intricate interactions within TFIIH. The network between XPB and p52 is expanded by interactions to p8 and in the human TFIIH model it was shown that L21 in p8, which causes TTD when mutated to a proline, is located at the HD2 interface of XPB (26). How the intricate network of protein interactions regulates XPB activity is, however, poorly understood so far.

In this study, we functionally dissected the regulatory events in XPB activation utilizing proteins from the eukaryotic fungus *Chaetomium thermophilum* as a model system. We solved the crystal structure of a p52/p8 complex and used this structure in combination with the current cryo-EM models to generate point mutations for the investigation of crucial p52/p8 interactions with XPB. We show that p52 not only acts as an activator of XPB but that also p8 in combination with p52 directly stimulates XPB’s ATPase activity further. Intriguingly, our data demonstrate that p52/p8 not only function as activators but at the same time repress the maximum activity that can be achieved when XPB is stimulated by DNA. This speed limiting effect can be observed in the absence of other core TFIIH components but also in the context of complete core TFIIH. Finally, we show that XPB’s ATPase activity does not directly lead to the translocase function of XPB. XPB requires the presence of all core TFIIH components to act as a translocase and the addition of XPA increases the processivity of the TFIIH translocase but not its ATPase activity. Our results suggest that XPB’s activity is tightly regulated at different levels to fine-tune its activity for the different processes in NER and transcription.

## MATERIALS AND METHODS

### Expression and purification

The genes encoding ctXPB, ctXPD, ctp62, ctp52, ctp44, ctp34, ctp8 and ctXPA were cloned from cDNA from *C. thermophilum*. The cDNA sequence of ctXPB was codon-optimized for expression in *Escherichia coli* (ATG:biosynthetics). CtXPB was inserted into both, the pBADM-11 vector (EMBL) containing an N-terminal hexa-histidine tag with a TEV cleavage site, and the pFastBac vector (Invitrogen) containing a C-terminal Twin-Strep tag and a 10×-histidine tag (The latter ctXPB was only used for the pull-down assays). Ctp44, ctp34, ctp8 and ctXPA were inserted individually into the pBADM-11 vector containing an N-terminal hexa-histidine tag with a TEV cleavage site. CtXPD was inserted into the pBADM-11 vector with or without an N-terminal hexa-histidine tag. Ctp62 and ctp52 were each inserted into the pETM-11 vector (EMBL) without tag. The constructs ctp52_1–321, ctp52_121–514 and ctXPB_60–345 were cloned utilizing the full length cDNA sequences via sequence and ligation independent cloning (30). For ctp52_121–514 a 28 amino acid long linker region of ctp52 (amino acid 322–349), that is not present in human p52, was replaced by a short linker (sequence SNGNG) via sequence and ligation independent cloning (30). The Walker A variants ctXPD_K48R and ctXPB_K392R as well as all ctp52 variants were generated via site directed mutagenesis (31). CtXPB from the pFastBac vector was expressed utilizing the bacculovirus system. Bacmids were prepared in SF21 cells in EX-CELL 420 medium (Sigma-Aldrich) at 27°C. The cell culture medium containing the viruses was harvested after approximately 72 h. Hi5 cells were grown in EX-CELL 405 medium (Sigma-Aldrich) at 27°C until a density of 0.5 × 10^6^ cells/ml was reached followed by transfection with 10% (v/v) virus containing medium. Protein expression was allowed for 72 h at 27°C. CtXPB from the pBADM-11 vector was expressed in Rosetta™ 2 (DE3) cells (Merck Millipore). CtXPD was expressed in ArcticExpress (DE3) RIL cells (Agilent). Ctp62/ctp44, ctp52/ctp8 as well as ctp52/ctp34 were co-expressed in BL21 CodonPlus (DE3) RIL cells (Agilent). Ctp8, ctp52_1–321, ctp52_121–514 and ctXPB_60–345 were separately expressed in BL21 CodonPlus (DE3) RIL cells. CtXPA was expressed in LOBSTR BL21 (DE3) RIL cells (Kerafast). The *E. coli* cells were grown at 37°C in either Lennox broth (XPB, p8, XPB_60–345, p52/p8, p52/p34, p52_121–514) or Terrific broth (XPB, XPD, p62/p44) medium (Carl Roth) to reach a final OD_600_ of 0.6 or 1.2, respectively. For the seleno-methionine containing variants of ctp52_1–321 and ctp52_121–514 the cells were grown in M9 minimal medium supplemented with L-seleno-methionine at 37°C to a final OD_600_ of 0.6 (32). When the final OD_600_ was reached, protein expression was induced by addition of 0.5 mM IPTG for pETM-11 vectors or 3.3 mM arabinose for pBADM-11 vectors accompanied by a temperature reduction to 15°C and protein expression was allowed over night. For expression of XPD, the temperature was reduced to 30°C once an OD_600_ of 0.6 was reached and the cells were allowed to grow until an OD_600_ of 1.2 was reached followed by induction and protein expression at 11°C.

For purification of the ctXPD/ctp44/ctp62 complex, ctp44 and ctp62 were co-purified via immobilized metal affinity chromatography (IMAC) using Ni IDA beads (Macherey-Nagel) followed by size exclusion chromatography (SEC) via a HiLoad 16/600 Superdex 200 prep grade column (Cytiva) with 20 mM Hepes pH 7.5, 250 mM NaCl, and 1 mM TCEP. The elution fractions containing ctp44/ctp62 were pooled and directly added to the cleared lysate of ctXPD. This mixture was subjected to another IMAC, followed by SEC and anion exchange chromatography (AEC). For SEC, the same column and buffer as above was used. For AEC the mixture was applied to a MonoQ 5/50 GL column (Cytiva), with buffers containing 20 mM Hepes pH 7.5, 50/1000 mM NaCl, and 1 mM TCEP. To obtain the ctp44/ctp62 complex in the absence of ctXPD, ctp44 and ctp62 were purified via IMAC and SEC as described above, followed by AEC using a MonoQ 5/50 GL column with the same buffers as for the ctXPD/ctp44/ctp62 complex. To obtain ctXPD, the protein was purified via IMAC, followed by SEC and AEC. The SEC buffer contained 20 mM Hepes pH 7.5, 150 mM NaCl, 5 mM MgCl_2_, and 1 mM TCEP. The AEC buffers contained 20 mM HEPES pH 7.5, 80/1000 mM NaCl, 5 mM MgCl_2_, and 1 mM TCEP. CtXPB (Hi5 cells) was purified using a 5 ml StrepTrap HP column (Cytiva) followed by SEC using a HiLoad 16/600 Superdex 200 prep grade column. For SEC, the buffer contained 20 mM Hepes pH 8.0, 200 mM NaCl and 1 mM TCEP. CtXPB (expressed in *E. coli*), ctXPB_60–345, ctp52/ctp34, ctp52/ctp8, ctp52_1–321, ctp52_121–514 and ctp8 were purified via IMAC (Ni TED or Ni IDA, Macherey-Nagel) and SEC using a HiLoad 16/600 Superdex 200 prep grade column (Cytiva). For ctXPB and ctp52/ctp34, the SEC buffer contained 20 mM HEPES pH 7.5, 250 mM NaCl, and 1 mM TCEP. For ctXPB_60–345, ctp52_1–321 and ctp52_121–514, the SEC buffer contained 20 mM Tris–HCl pH 7.5 and 250 mM NaCl. For ctp52/ctp8 and ctp8 the SEC buffer contained 20 mM Hepes pH 8 and 375 mM NaCl. All variants were expressed and purified as described for the wild type proteins. In case of ctp8, two versions were used. For the co-crystallization with ctp52_121–514, ctp8 containing an N-terminal hexa-histidine tag and a TEV cleavage site was used. For the other studies, the tag was removed by incubation with TEV protease after IMAC and the protein solution was subjected to another IMAC to remove the TEV protease and uncut protein prior to SEC. CtXPA was purified via IMAC and AEC using a MonoQ 5/50 GL column with buffers containing 20 mM Tris–HCl pH 8, 50/1000 mM NaCl and 2 mM TCEP.

All proteins were concentrated to 50–1000 μM, flash frozen in liquid nitrogen, and stored at –80°C.

### Crystallization

Crystallization was performed via the vapour diffusion method at 20°C. For ctp52_1–321, a seleno-methionine derivative was crystallized at concentrations of 4–7 mg/ml. The reservoir solution consisted of 100 mM calcium acetate, 12.5% (w/v) PEG 8000, and 100 mM HEPES pH 7.5. For ctp52_121–514, a seleno-methionine derivative was crystallized at protein concentrations of 5–6 mg/ml. The reservoir solution consisted of 7.5% (w/v) PEG 4000 and 100 mM HEPES pH 7.0. For the ctp52_121–514/ctp8 complex, ctp52_121–514 at concentrations of 5–6 mg/ml was incubated for 1 h at 4°C with a twofold excess of ctp8 prior to crystallization. The reservoir solution consisted of 150 mM ammonium chloride, 15% (w/v) PEG 4000, and 100 mM Tris–HCl pH 8.5.

### Structure solution and refinement

X-ray diffraction data were processed with XDS (33). Diffraction data for the ctp52_121–514/ctp8 complex were anisotropy-corrected using the STARANISO server (34). Initial phases were obtained using SHARP (35) utilizing the anomalous signal of the dataset from the seleno-methionine derivate of ctp52_121–514. The resulting electron density map was used to build an initial model of ctp52_121–514. This model was used to solve the phase problem for the ctp52_1–321 and ctp52_121–514/ctp8 datasets via molecular replacement with Phaser (36). The models for ctp52_1–321 and ctp52_121–514/ctp8 were manually completed and corrected with Coot (37). For the latter, the available structure of a Tfb5/Tfb2 minimal complex (PDB code: 3DOM), was used as additional template for manual model building. The ctp52_1–321 structure was refined with REFMAC5 (38) using automated twin refinement. The ctp52_121–514/ctp8 structure was refined with BUSTER (39).

### ATPase assay

CtXPB’s ATPase activity was quantified utilizing an *in vitro* ATPase assay in which ATP consumption is coupled to the oxidation of NADH via pyruvate kinase and lactate dehydrogenase activities. Activities were measured at 30°C in 100 μl solution containing 1.3 U pyruvate kinase, 1.9 U lactate dehydrogenase (PK/LDH, Sigma), 1.6 mM phosphoenolpyruvate, 0.24 mM NADH, 10 mM KCl, 2.5 mM MgCl_2_, 1 mM TCEP, and 20 mM Tris–HCl pH 8. The assay was carried out under saturating concentrations of ATP using ctXPB wild type and ctXPB_K392R at a concentration of 250 nM. Ctp52, ctp52 variants, ctp8 and ctp34 were added in a twofold molar excess to ctXPB. When core ctTFIIH was assembled, all subunits were present at an equimolar ratio and a final concentration of 250 nM. CtXPA was added in a 4:1 stoichiometric ratio to ctXPB or core ctTFIIH. If not stated otherwise, double stranded (dsDNA) (fw: 5′-AGCTACCATGCCTGCACGAATTAAGCAATTCGTAATCATGGTCATAGC-3′; rv: 5′-GCTATGACCATGATTACGAATTGCTTAATTCGTGCAGGCATGGTAGCT-3′) was used at a final concentration of 125 nM for ctXPB or 1 μM for core ctTFIIH. The sample was preincubated until a stable base line was achieved. Enzyme catalysis was initiated by the addition of 2.5 mM ATP. The activity profiles were measured at 340 nm using a Clariostar plate reader (BMG Labtech) and 384-well F-bottom μClear™ microplates (Greiner Bio-One). Initial velocities were recorded and ATP consumption was determined using the molar extinction coefficient of NADH. Curves were fitted with GraphPad Prism. All measurements were carried out at least in triplicates, using at least two independently purified batches of the two helicases XPB and XPD as well as their Walker A variants. Example time courses are shown in Supplementary Figure S1A. SDS PAGE analyses of the complexes used in this study are shown in Supplementary Figure S2.

### Triplex displacement assay

Core ctTFIIH dsDNA-translocase activity was detected using a well established triplex disruption assay (25,28). DsDNA-translocase activity was measured by displacement of a fluorescently labeled triplex forming oligonucleotide (TFO) from a triple helix DNA substrate. The assay was carried out at 30°C in 50 μl solution containing 120 mM KCl, 8 mM MgCl_2_, 16 mM HEPES–KOH pH 8, 4% (v/v) glycerol, 0.8 mM TCEP, 2.2 mM phosphoenolpyruvate, 1.8 U pyruvate kinase (Sigma-Aldrich) and 150 nM triplex DNA. The triplex DNA consists of a 52 bp dsDNA strand with a 5′ dabcyl label (fw: 5′-GTCTTCTTTTAAACACTATCTTCCTGCTCATTTCTTTCTTCTTTCTTTTCTT-3′; rv: 5′-Dabcyl-AAGAAAAGAAAGAAGAAAGAAATGAGCAGGAAGATAGTGTTTAAAAGAAGAC-3′) and a 5′ Cy3-tagged TFO (5′-Cy3-TTCTTTTCTTTCTTCTTTCTTT-3′). The triplex DNA was annealed as previously described (28). Correct triplex formation was confirmed via native PAGE. The baseline was recorded for 10 to 15 minutes prior to addition of 2 mM ATP. TFO displacement was measured for 60 min at an excitation wavelength of 520–540 nm and an emission wavelength of 590–620 nm with a gain of 1900 using a Clariostar plate reader (BMG LABTECH) in 384-well F-bottom FLUOTRAC™ high binding microplates (Greiner Bio-One). When core ctTFIIH was assembled, all subunits were present in equimolar amounts with a final concentration of 500 nM. CtXPA was added at a fourfold molar excess to core ctTFIIH or ctXPB. For the minimal XPB complex, ctp52, ctp8 and ctp34 were added at a twofold molar excess to ctXPB (500 nM). The release of Cy3-tagged TFO corresponds to the slope of increasing fluorescence. Curves were fitted with GraphPad Prism. All measurements were carried out in at least eleven replicates using two independently purified batches of the two helicases XPB and XPD as well as their Walker A variants. Example time courses are shown in Supplementary Figure S1B. SDS PAGE analyses of the complexes used in this study are shown in Supplementary Figure S2.

### Pull down assay

10 μl Strep-Tactin Sepharose beads (IBA) were equillibrated with 100 μl buffer containing 150 mM NaCl, 1 mM TCEP and 20 mM HEPES pH 7.5. 2 μM Twin-Strep-tagged ctXPB was added and incubated for 45 minutes with the beads. 16 μM ctp52/ctp8 or ctp52_E359K/ctp8 were added and incubated for another 45 minutes. As control, 16 μM ctp52/ctp8 was incubated with the beads in the absence of ctXPB. Unbound protein was washed off with buffer and bound protein was eluted in 20 μl buffer supplemented with SDS loading dye and boiled for 5 min at 95°C. Elution fractions were analysed by SDS PAGE. The intensities of the ctp52/p8 gel bands were quantified using ImageJ and graphs were generated via GraphPad Prism. Three technical replicates were performed.

### Fluorescence polarization measurements

DNA binding was analysed by fluorescence polarization employing a duplex DNA with a Cy3 label (fw: 5′-Cy3-AGCTACCATGCCTGCACGAATTAAGCAATTCGTAATCATGGTCATAGC-3′ rv: 5′-GCTATGACCATGATTACGAATTGCTTAATTCGTGCAGGCATGGTAGCT-3′). Assays were carried out in 50 μl solution with 20 mM HEPES pH 7.5, 10 mM KCl, 2.5 mM MgCl_2_, 1 mM TCEP, and 6 nM DNA at room temperature. CtXPB was used at concentrations of 0.5–500 nM as indicated. Ctp52/ctp8 was added at a 2:1 stoichiometric ratio to ctXPB. Fluorescence polarization was detected at an excitation wavelength of 540 nM and an emission wavelength of 590 nM with a Clariostar plate reader (BMG LABTECH) using 384-well F-bottom Fluotrac high binding microplates (Greiner Bio-One). The gain was adjusted to a well containing buffer and DNA only. Curves were fitted with GraphPad Prism. Three technical replicates were performed.

### CD spectroscopy

Circular dichroism (CD) spectra were recorded under constant nitrogen flush using a Jasco J-810 spectropolarimeter over 190–260 nm. Measurements were performed at 20°C in eight scans with a speed of 50 nm/min and a band width of 2 nm. The proteins were diluted to 4 μM in a buffer containing 19 mM dipotassium phosphate and 1.2 mM monopotassium phosphate at pH 8. All samples were centrifuged for 20 minutes at 30 000 g prior to the measurement. The buffer spectrum was used as a baseline and subtracted from all protein spectra.

### Native PAGE

Native PAGE was performed using Tris/glycine gels consisting of 12.5 mM Tris, 96 mM glycine pH 8.9, 12% (v/v) ROTIPHORESE^®^ Gel 30 (37.5:1) (Roth), 0.07% (w/v) APS, and 0.1% (v/v) TEMED. All samples contained 10 μM protein in 4 mM Tris, 16 mM HEPES pH 8, 350 mM NaCl. The samples were incubated on ice for 1 h, supplemented with 5× loading dye (0.08% (w/v) Ponceau S and 50% (v/v) glycerol) and loaded on the gel. Electrophoresis was performed at 4°C in a buffer containing 12.5 mM Tris and 96 mM glycine pH 8.9 for 90 min at 100 V followed by 3:45 h at 200 V. Protein bands were visualized by Coomassie G-250 staining.

To confirm correct triplex DNA annealing, native PAGE was performed using Tris-acetate gels consisting of 40 mM Tris pH 5.5, 40 mM acetate, 5 mM magnesium acetate, 1 mM MgCl_2_, 8% (v/v) ROTIPHORESE® Gel 40 (29:1) (Roth), 0.1% (w/v) APS and 0.1% (v/v) TEMED. All samples contained 1 μM triplex DNA supplemented with 5 x loading dye. Electrophoresis was performed in a buffer containing 40 mM Tris pH 5.5, 40 mM acetate, 5 mM magnesium acetate, and 1 mM MgCl_2_ for 55 min at 100 V. DNA was visualized using a Pharos FX™ Plus imager (Bio-Rad).

## RESULTS

### The ctp52/ctp8 structure

**Figure 1.**
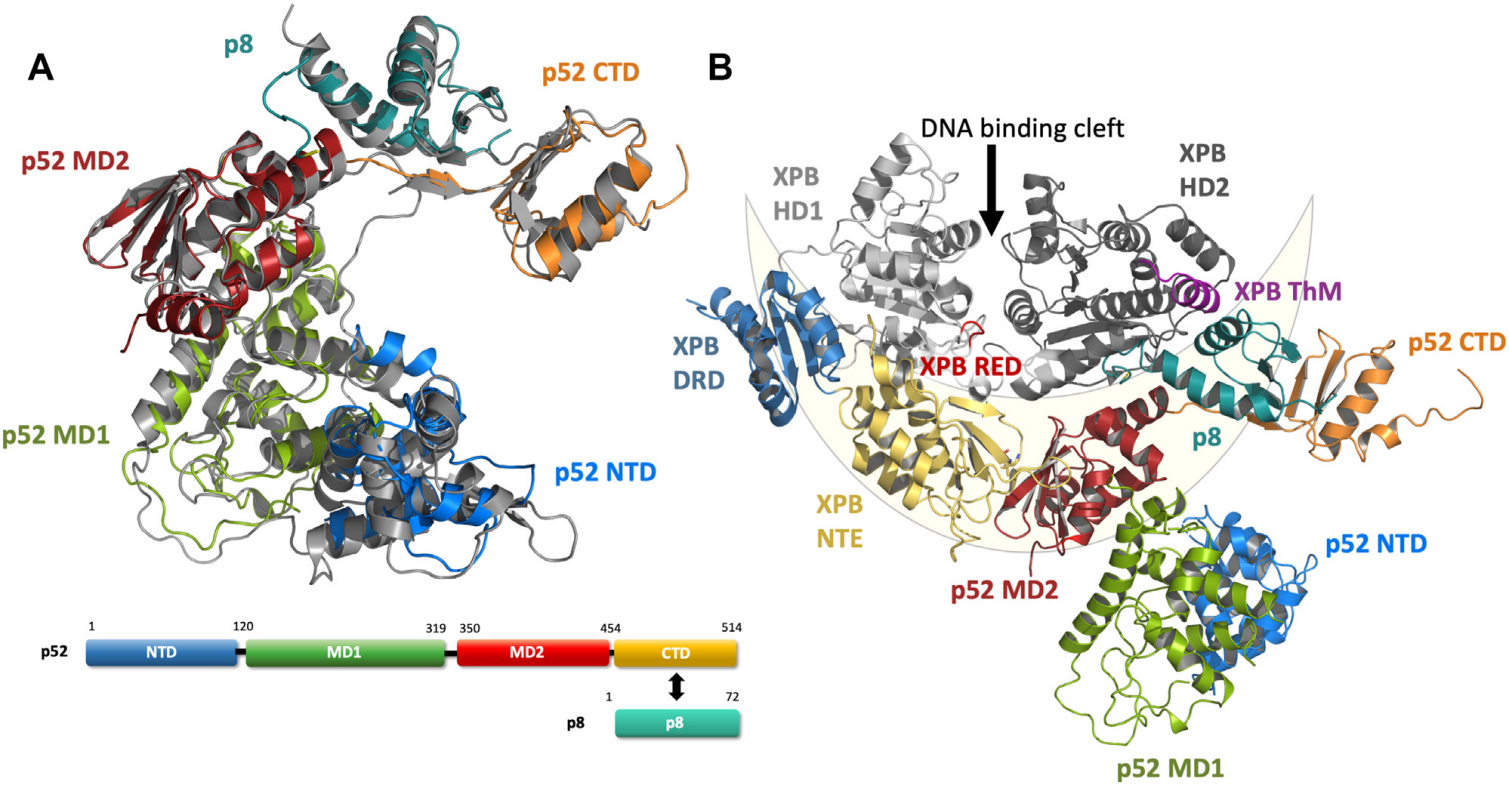
Structure and domain architecture of ctp52 and ctp8. (**A**) Structure and domain architecture of full length ctp52 and ctp8. Ctp52 is comprised of four domains: NTD (blue), MD1 (green), MD2 (red) and CTD (orange). Ctp8 is shown in mint green. Ctp8 and ctp52 CTD adopt the same fold. The domains have been rearranged to fit to the p52 conformation found in the cryo-EM structure of human TFIIH (shown in grey). (**B**) Ctp52 and ctp8 modelled into the TFIIH cryo-EM structure from Greber *et al.* (26) from which only XPB, p52 and p8 are shown. The lunate-like ring that encircles the XPB helicase domains is schematically depicted in light yellow.

Several cryo-EM structures of TFIIH have recently been published shedding light on the molecular architecture of the core TFIIH components including the important p52/p8 complex (26–29). However, no full atomic model of p52, the major regulator of the XPB enzyme, alone or in complex with p8 beyond a resolution of 3.5 Å was available so far. We pursued the structural characterization of p52 from the fungal model organism *Chaetomium thermophilum* (ctp52) using x-ray crystallography. Ctp52 shares 35% and 39% sequence identity with the human p52 and yeast Tfb2 sequences, respectively (Supplementary Figure S3). We solved two crystal structures of ctp52. The first structure encompasses amino acids 1–321 (ctp52_1–321) and was solved at a resolution of 2.8 Å (Table 1, Supplementary Figure S4A). The second structure encompasses the C-terminal part starting at amino acid 121 (ctp52_121–514) and was solved in complex with ctp8 at a resolution of 2.7 Å (Table 1, Supplementary Figure S4B). In the latter structure a 28 amino acid long linker region of ctp52 (amino acids (aa) 322–349), that is not present in human p52, was replaced by a short linker (sequence SNGNG). Due to the overlap of both structures, we were able to generate a full structural model of ctp52 at atomic resolution (Figure 1A, Supplementary Figure S4C). The model shows that ctp52 is organized into four distinct domains, an N-terminal domain (NTD; aa 1–120), two middle domains (MD1; aa 121–319 and MD2; aa 350–454), and a C-terminal domain (CTD; aa 455–514), that interacts with p8 (40). The linker region that was replaced in ctp52_121–514, bridges MD1 with MD2 and could not be observed in our structure due to its flexibility. The ctp52_1–321 model comprises the NTD and MD1 domains and the ctp52_121–514 model the MD1, MD2, and CTD domains. Both crystal structures were superimposed via the MD1 domain (rmsd of 0.87 Å) thereby generating the full-length ctp52 model.

**Table 1.** Data collection and refinement statistics

	p52_1–321	p52_121–514/p8	p52_121–514 (ano)
**Data collection**			
Wavelength (Å)	0.97973	0.96770	0.97977
Spacegroup	*P* 3_1_	*P* 2_1_	*P* 1
Unit cell parameters			
*a*, *b*, *c* (Å)	104.3, 104.3, 165.0	74.2, 86.0, 92.2	60.5, 83.2, 86.0
α, β, γ (°)	90, 90, 120	90, 94.9, 90	82.7, 80.0, 77.5
Resolution range (Å)	46.97–2.80	19.92–2.68	49.16–2.60
	(2.89–2.80)	(2.95–2.68)	(2.69–2.60)
Unique reflections	49 518 (4628)	17 307 (865)	48 667 (4455)
*R*_merge_ (%)	16.2 (282.9)	28.0 (142.9)	20.1 (134.2)
*R*_pim_ (%)	5.3 (90.1)	14.0 (71.5)	8.1 (53.9)
*I*/σ*I*	10.7 (1.0)	5.7 (1.2)	9.4 (1.5)
CC (1/2)	0.997 (0.325)	0.983 (0.318)	0.995 (0.494)
Multiplicity	10.4 (10.8)	4.9 (4.8)	7.0 (7.1)
Completeness (%)			
spherical	100.0 (100.0)	53.4 (10.8)	98.7 (98.0)
ellipsoidal	-	88.7 (49.5)	-
**Refinement**			
Resolution range (Å)	46.97–2.80	19.92–2.68	
Unique reflections	46 992	17 099	
Number of atoms	8572	5606	
*R*_work_ (%)	18.6	21.6	
*R*_free_ (%)	20.9	24.0	
Mean *B*-factor (Å^2^)	88.9	53.2	
RMS deviations			
Bond lengths (Å)	0.0054	0.008	
Bond angles (°)	1.4375	0.95	
Ramachandran statistics (%)			
Favored	81.17	95.20	
Allowed	15.96	3.93	
Outliers	2.87	0.87	

Values in parentheses correspond to highest resolution shell.

The ctp52 structure is predominantly α-helical and consists of 22 α-helices and 15 β-strands (Figure 1A, Supplementary Figure S4C). None of the newly identified domains present in ctp52 (NTD, MD1 and MD2) displays significant structural homologies to any known protein domains. The interaction between ctp52 and ctp8 is mediated by β-strands 12–15 of p52 and β-strands 1 to 3 of p8 and can be readily compared to the crystal structure of the minimal yeast Tfb5/Tfb2 complex (40) (PDB code: 3DOM) with an rmsd of 1.10 Å for the CTD of ctp52 and an rmsd of 0.97 Å for ctp8 (PDBsum).

A superposition of the ctp52/ctp8 structure with the cryo-EM model of human p52/p8 within TFIIH (26) shows that the MD2 domain was found to be oriented differently compared to our model (Supplementary Figure S4D). While the antiparallel β-sheet of MD2 (β-strands 8–11) is pointing towards MD1 and is involved in the formation of the dimer interface in our ctp52_121–514/ctp8 structure (Supplementary Figure S4B), it is pointing towards the N-terminal extension (NTE) of XPB in the TFIIH model (Figure 1B). This is most likely due to the different conditions and interactions in the crystallization experiments compared to the cryo-EM experiment. However, after the rearrangement of the individual ctp52 domains according to the model of human p52/p8 embedded in TFIIH an overall rsmd of 1.6 Å was obtained, indicating a high degree of structural similarity. Thus, we generated a ctp52/ctp8/XPB hybrid model (Figure 1B), which contains the high resolution p52/p8 information obtained from our crystal structure embedded in the core TFIIH arrangement as observed in the cryo-EM structure of human TFIIH (6NMI, (26)).

The main and most relevant interaction between the MD2 of p52 and the NTE of XPB seems to be mediated by an orthogonal packing of their respective β-sheets (Figure 1B). Furthermore, MD2 of p52 is also interacting with the second helicase domain, HD2, of XPB in the ctp52/ctp8/XPB hybrid model (Figure 1B). This interaction involves twelve residues of the MD2 domain located mainly in α-helix 20 of p52 and the β-turns that connect β-strand 8 to 11. The other side of the interface is formed by eleven residues of XPB’s HD2. Based on the cryo-EM structure of human TFIIH (PDB code: 6NMI), we observed that both interfaces combined comprise an approximate surface area of 1580 Å^2^. Importantly, an extension of the interface is achieved through the presence of p8 which also interacts with the HD2 of XPB thereby providing an additional surface area of approximately 516 Å^2^.

The hybrid structure illustrates that four entities including p8 and the MD2 of p52, together with the NTE and the DRD domains of XPB form a lunate-like ring (Figure 1B), which embraces the two RecA like domains of XPB from one side, while the DNA approaches the HD domains from the other side (27,28). DNA binding, a functional Walker A motif and an intact RED (arginine-glutamate-aspartate) motif as well as the thumb-like motif (ThM) seem to be prerequisites for the activity of XPB during NER (4,6,9). In addition, the lunate-like ring may be required to position the two RecA like domains of XPB in a conformation which stimulates its ATPase activity. Interestingly, p8 directly interacts with the ThM motif of XPB. Due to the importance of XPB’s ATPase activity in transcription and DNA repair, we pursued further studies to decipher the functional influence of the individual components and interactions of this intricate network.

### The ATPase activity of ctXPB is sequentially stimulated by ctp52 and ctp8

**Figure 2.**
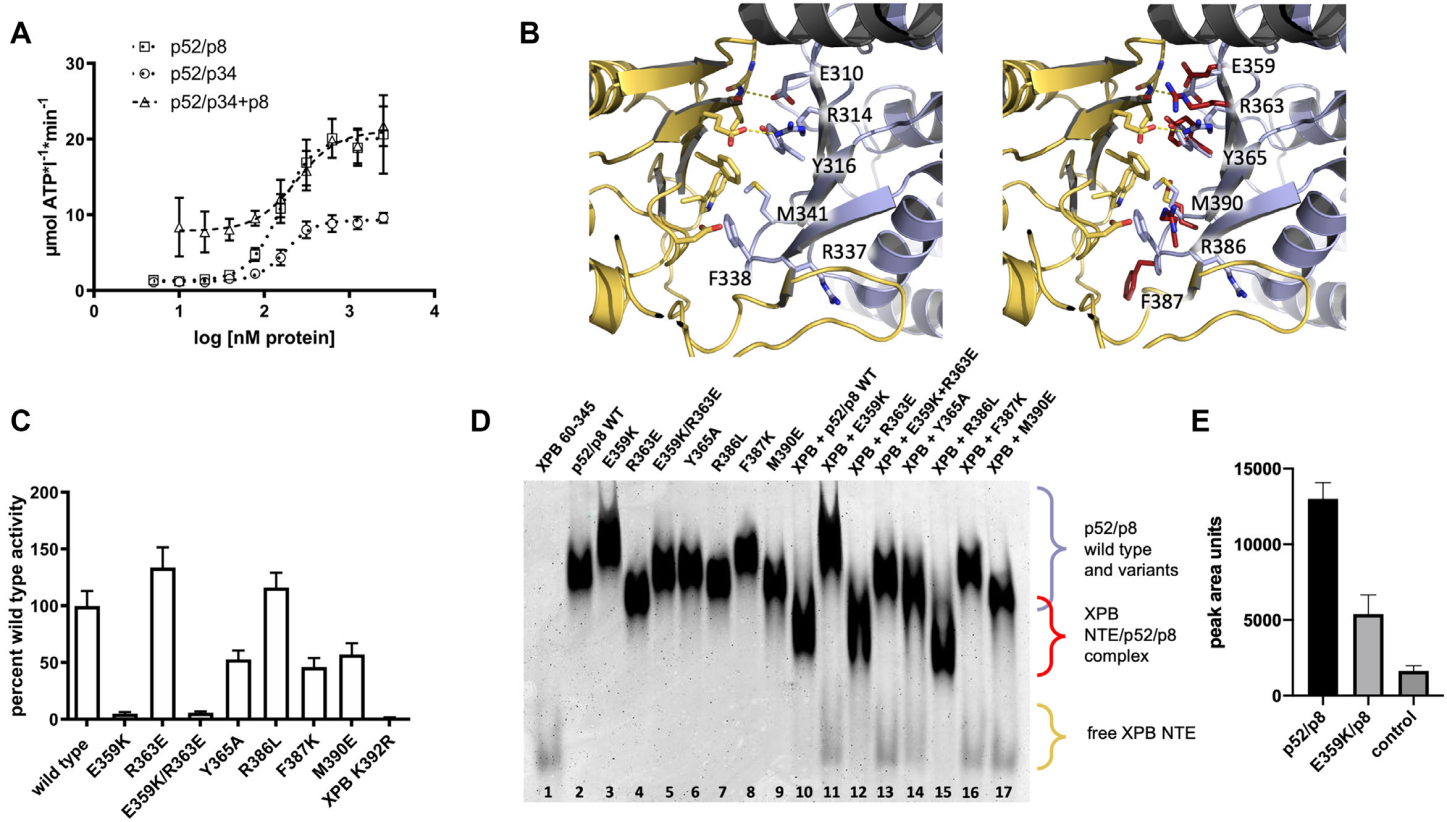
Activation of XPB’s ATPase by p52/p8 and interaction studies of ctp52 MD2 and ctXPB NTE. (**A**) ATPase activity profile of ctXPB activated by increasing amounts of different ctp52 complexes. Ctp52 complexes were titrated to a fixed amount of ctXPB. The x-axis shows the logarithmic concentration of the respective ctp52 complex. The y-axis shows the NADH-consumption-derived ATP turnover rate in μmol ATP per liter per minute. *N* ≥ 3. (**B**) Structural insight into the human p52 (light blue) – XPB (yellow) interface taken from 6NMI. On the left hand side, key residues in the interface are labeled. On the right hand side, the corresponding residues in ctp52 (red) are superimposed to the human counterparts and labeled. The depicted ctp52 residues were mutated and analysed with respect to their influence on ctXPB’s ATPase activity (**C**) and in terms of their interaction with the ctXPB NTE (**D**). (**C**) Effect of ctp52/ctp8 wild type and variants on the ATPase activity of ctXPB. *N* = 8. (**D**) Native PAGE analysis of the interaction between the ctp52/ctp8 variants and ctXPB NTE (ctXPB 60–345). Individual proteins are shown in lanes 1–9. Ctp52 wild type and variants in complex with ctp8 and ctXPB NTE are shown in lanes 10–17. (**E**) Pull-down of ctp52/ctp8 and ctp52_E359K/ctp8 with Streptavidin-tagged ctXPB as bait. The pull down fractions were loaded on SDS PAGEs and the SDS PAGE band strength of p52/p8 wild type and E359K was analysed with ImageJ to quantify the interaction capability with full length XPB. Mean values with SD were derived with GraphPad Prism. To exclude unspecific binding to the beads, p52/p8 was loaded as a control to the beads without XPB. *N* = 3.

To investigate which components are necessary for the full activation of XPB, we used the proteins from *C. thermophilum* due to their superior stability. It was initially shown by Egly and colleagues, that p52 stimulates the ATPase activity of XPB and that p8 could aid in this process (6,41). Hence, we aimed to analyze the activation mechanism. To this end, we first performed titration experiments using a co-purified ctp52/ctp34 complex. Ctp34 was used to enhance the stability of ctp52 and to ensure a native ctp52 conformation in accordance with the composition in TFIIH. Ctp52/ctp34 activates ctXPB in a concentration dependent manner, reaching a *V*_max_ of 9.3 μmol ATP*l^−1^*min^−1^ and an EC_50_ value of 178 nM (Figure 2A, open circles). When ctp8 is added to the ctXPB/ctp52/ctp34 complex, the basal activity level is 7.9 μmol ATP*l^−1^*min^−1^ and reaches a maximum of 21.1 μmol ATP*l^−1^*min^−1^ with an EC_50_ value of 238 nM (Figure 2A, open triangles) indicating that ctp8 is directly able to further enhance the ctp52-mediated activation of ctXPB. We then investigated whether a co-purified ctp52/ctp8 complex alone is able to activate ctXPB. Here, we observed a full activation with 20.1 μmol ATP*l^−1^*min^−1^ and an EC_50_ value of 153 nM (Figure 2A, open squares), showing that ctp52/ctp8 is sufficient for ctXPB activation and that ctp34 does not alter the activation cascade. Example time courses for the ATPase measurements are shown in Supplementary Figure S1A and the SDS PAGE analysis confirms the integrity and purity of the used complexes (Supplementary Figure S2).

Based on our ctp52 crystal structure modelled into the most recent cryo-EM map we were able to identify conserved ctp52 residues in the MD2 domain that interact with the ctXPB NTE. We chose these residues for functional mutagenesis studies since it was also shown that some of these residues (E359, R363) led to phenotypic consequences when they are mutated (41). We therefore generated the following ctp52 variants: E359K, R363E, E359K/R363E, Y365A, R386L, F387K and M390E (Figure 2B). All residues are located in a β-sheet that interacts with a similar motif within the ctXPB NTE. We thus targeted the entire interface from the ctp52 side ranging from a hydrophobic area comprised of Y365, F387 and M390 to the charge complementary patch constituted by E359 and R363. R386 displays different side chain conformations in the cryo-EM structure compared to the crystal structure and was thus chosen to assess which conformation is relevant (Figure 2B). Additionally, Smurnyy *et al.* observed that this residue leads to triptolide resistance in mammalian cells when mutated to a leucine, hinting towards a crucial role in the activation of XPB’s ATPase (42). All ctp52 variants were subjected to CD spectroscopy to ensure that no misfolded variants were used in the analysis (Supplementary Figure S5). Based on the observation that ctp52/ctp8 leads to a higher activation of XPB’s ATPase, than ctp52 alone, we investigated whether those variants were still able to activate ctXPB in the ctp52/ctp8 context (Figure 2C). The most severe impact on ctXPB activation was observed for the E359K variant that was only able to activate ctXPB to 5% of the wild type level, whereas the adjacent R363E variant had no effect and displays 134% of wild type activity. In line with the result for the single E359K variant, the E359K/R363E variant displays a comparable decrease to 6% activation. The Y365A, F387K, and M390E variants all display a medium phenotype with an activation profile of 53%, 46% and 57%, respectively, indicating at least partial activation. Mutation of R386 to a leucine (R386L) has no effect showing an activation of 116% compared to the wild type proteins. To further investigate whether these variants still interact with the NTE of ctXPB we performed native PAGE analysis using the ctp52 variants in complex with ctp8 and the NTE of ctXPB comprising residues 60–345 (ctXPB NTE, Figure 2D). All ctp52/ctp8 variant complexes form a single band in the native PAGE, however, due to the different charge properties of the variants they are located at slightly different heights (Figure 2D, lanes 3–9) compared to the wild type complex (Figure 2D, lane 2). Combining ctXPB NTE with wild type ctp52/ctp8 clearly leads to complex formation (Figure 2D, lane 10), whereas the variants that displayed reduced activation capabilities in Figure 2C (E359K, R363E/E359K, F387K, and M390E) also show no interaction in the native PAGE experiment (Figure 2D, lanes 11, 13, 16–17). Variant Y365A, which displayed a partial activation of ctXPB’s ATPase (Figure 2C), shows also a partial interaction in the native PAGE (Figure 2D, lane 14) indicating that the interface is affected by the mutation but binding is only partially disrupted. The ctp52/ctp8 variants that were able to activate ctXPB’s ATPase like wild type (R363E and R386L, Figure 2C) were capable of forming a complex with ctXPB NTE (Figure 2D, lane 12 and 15).

Since the cryo-EM structure of holo-TFIIH suggests the presence of a second XPB/p52 interface located in the HD2 domain of XPB and the MD2 of p52 (Figure 1B) (26) we analysed the E359K variant towards its interaction with full length ctXPB. Albeit decreased, we could indeed observe significant complex formation (Figure 2E). This result suggests that disrupting one of the interfaces is sufficient to prevent p52/p8 mediated XPB activation, whereas complex formation is partially maintained.

### ctp52/ctp8 limits ctXPB DNA dependent ATPase activity

**Figure 3.**
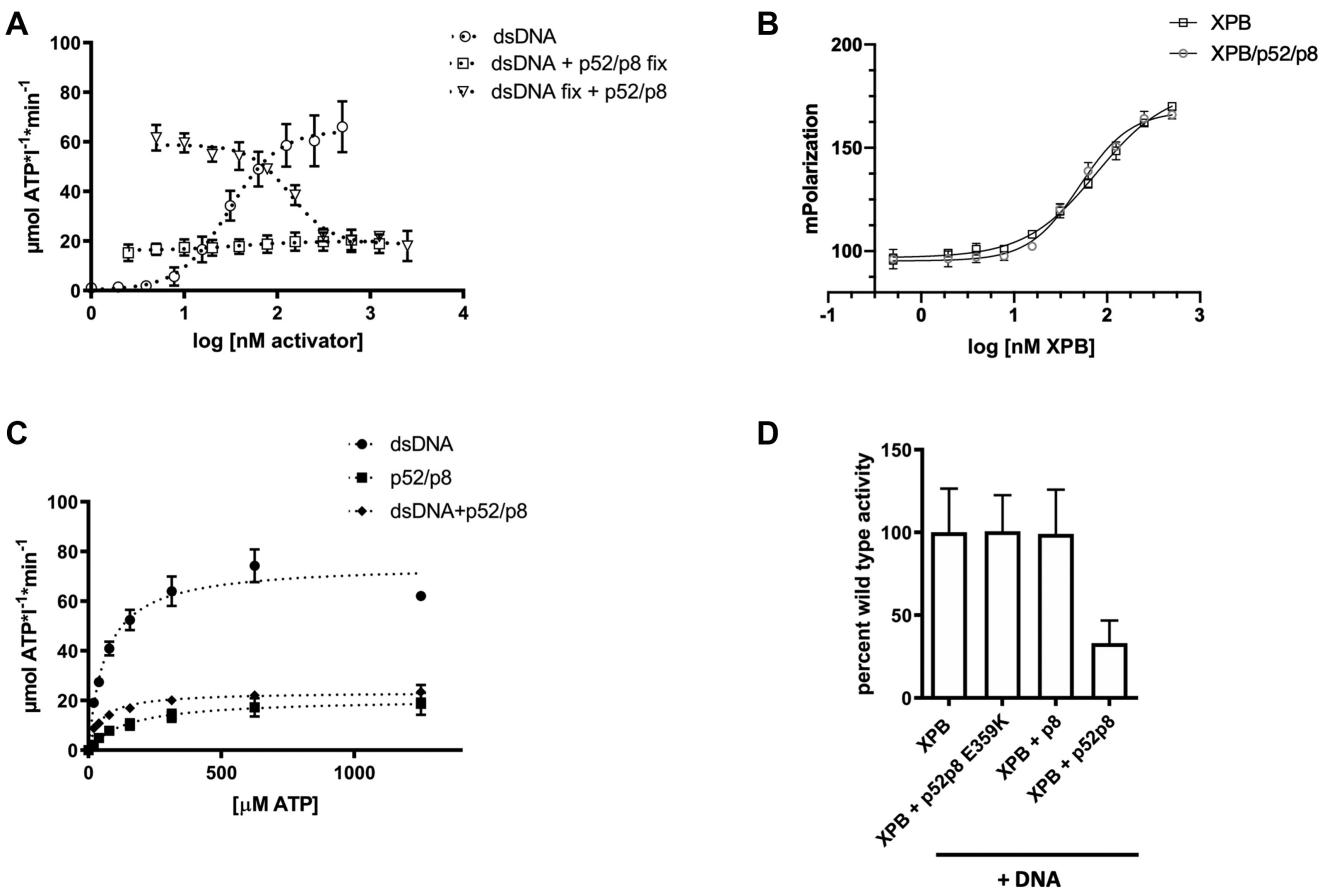
Influence of DNA and ATP binding on ctXPB’s ATPase activity. (**A**) Influence of dsDNA and ctp52/ctp8 on the ATPase activity of ctXPB. Increasing amounts of dsDNA were titrated onto fixed concentrations of ctXPB (open circles) and ctXPB with ctp52/ctp8 (open squares). Increasing amounts of ctp52/ctp8 were titrated onto fixed concentrations of ctXPB plus dsDNA (open triangles). The x-axis shows the logarithmic concentration of the respective activator complex that was titrated. The y-axis shows the NADH-consumption-derived ATP turnover rate in μmol ATP per liter per minute. *N* ≥ 4. (**B**) Fluorescence polarization measurements to investigate the influence of ctp52/ctp8 on ctXPB’s dsDNA binding ability. Increasing amounts of ctXPB (open squares) and ctXPB with ctp52/ctp8 (open circles) were titrated onto fixed amounts of fluorescently labeled dsDNA. The calculated k_D_ values are listed in Table 2. *N* = 3. (**C**) ATPase activity profile with Michaelis Menten kinetics. Increasing amounts of substrate (ATP) were titrated onto different complexes of ctXPB with its activators dsDNA (circles), ctp52/ctp8 (squares) and dsDNA plus ctp52/ctp8 (rhombs). The x-axis shows the concentration of the respective activator complex. The y-axis shows the NADH-consumption-derived ATP turnover rate in μmol ATP per liter per minute. *V*_max_, *K*_M_, *k*_cat_ and *k*_cat_/*K*_M_ values for the different ctXPB activation complexes are listed in Table 3. *N* ≥ 3. (**D**) ATPase activity of ctXPB in the presence of dsDNA with or without different ctp52/ctp8 complexes. *N* ≥ 8.

We next aimed to investigate the influence of DNA binding on the ATPase activity of ctXPB. In the sole presence of double stranded DNA (dsDNA) the activity of ctXPB reached a *V*_max_ of 64.6 μmol ATP*l^−1^*min^−1^ (Figure 3A, open circles). Interestingly, this is about three times higher than the activation of ctXPB via ctp52/ctp8 (See open squares in Figure 2A for comparison). Surprisingly, in the presence of ctp52/ctp8 and dsDNA the induced ATPase activity is limited to 19.9 μmol ATP*l^−1^*min^−1^ (Figure 3A, open squares) which corresponds well to the activity achieved with ctp52/ctp8 in the absence of dsDNA (Figure 2A, open squares). This result indicates that ctp52/ctp8 negatively controls the dsDNA-dependent activation of XPB, which is illustrated further when we titrated increasing amounts of ctp52/ctp8 to an equimolar ctXPB/DNA complex (Figure 3A, open triangles). At 125 nM, dsDNA ctXPB is already completely activated with an activity of 58.8 μmol ATP*l^−1^*min^−1^. This activity decreases in a ctp52/ctp8 concentration dependent manner until it reaches again 18.9 μmol ATP*l^−1^*min^−1^ with an EC_50_ value of 138 nM indicating a high binding affinity between ctXPB and ctp52/ctp8 and once an equimolar complex is formed, downregulation is complete. To further decipher the mechanism of activity limitation we investigated whether the binding of ctp52/ctp8 influences the DNA binding capacity of ctXPB. We therefore pursued fluorescence polarization measurements comparing ctXPB alone to the ctXPB/ctp52/ctp8 complex in the presence of DNA (Figure 3B and Table 2). The obtained k_D_ values were 77 nM for ctXPB and 51 nM for the ctXPB/ctp52/ctp8 complex. These similar values indicate that there is no major effect on dsDNA binding caused by the presence of ctp52/ctp8. Since dsDNA binding did not seem to be affected, we evaluated whether ATP binding is altered by the presence of ctp52/ctp8. Michaelis-Menten experiments to assess the apparent *K*_M_ for ATP (Figure 3C and Table 3) led to *K*_M_ values of 62.9 μM (ctXPB/dsDNA), 140.0 μM (ctXPB/ctp52/ctp8), and 45.0 μM (ctXPB/ctp52/ctp8 with excess dsDNA). These values indicate a positive effect on the *K*_M_ for ATP in the presence of dsDNA. However, the corresponding *V*_max_ value of ctXPB with excess dsDNA (74.9 μmol ATP*l^−1^*min^−1^) is around three times higher than the *V*_max_ of ctXPB/ctp52/ctp8/dsDNA (23.4 μmol ATP*l^−1^*min^−1^). The latter value fits well with the *V*_max_ of ctXPB/ctp52/ctp8 without dsDNA (20.7 μmol ATP*l^−1^*min^−1^) indicating that ctp52/ctp8 predominantly affects the *V*_max_ of the ctXPB/dsDNA complex. In addition, we calculated the *k*_cat_/*K*_M_ ratios for ctXPB/ctp52/ctp8 and ctXPB/ctp52/ctp8/dsDNA with 0.7 μM ATP*min^−1^ and 1.8 μM ATP*min^−1^, respectively. Both are significantly lower than the *k*_cat_/*K*_M_ ratio of the ctXPB/dsDNA complex with 4.8 μM ATP*min^−1^, indicating that also the catalytic efficiency is significantly decreased by p52/p8 when dsDNA is bound to XPB.

**Table 2.** *K*_D_ values for XPB calculated from fluorescence polarization measurements in Figure 3B

Complex	*k*_D_ (nM XPB)
XPB/DNA	77
XPB/p52/p8/DNA	51

**Table 3.** Kinetic parameters for ATP calculated from ATPase measurements in Figure 3C

Complex	*K*_M_ (μM ATP)	*V*_max_ (μmol ATP*l^−1^*min^−1^)	*k*_cat_ (1*min^−1^)	*k*_cat_/*K*_M_ (μM ATP*min^−1^)
XPB/dsDNA	62.9	74.9	299.6	4.8
XPB/p52/p8	140	23.4	93.6	0.7
XPB/dsDNA/p52/p8	45	20.7	82.8	1.8

**Figure 4.**
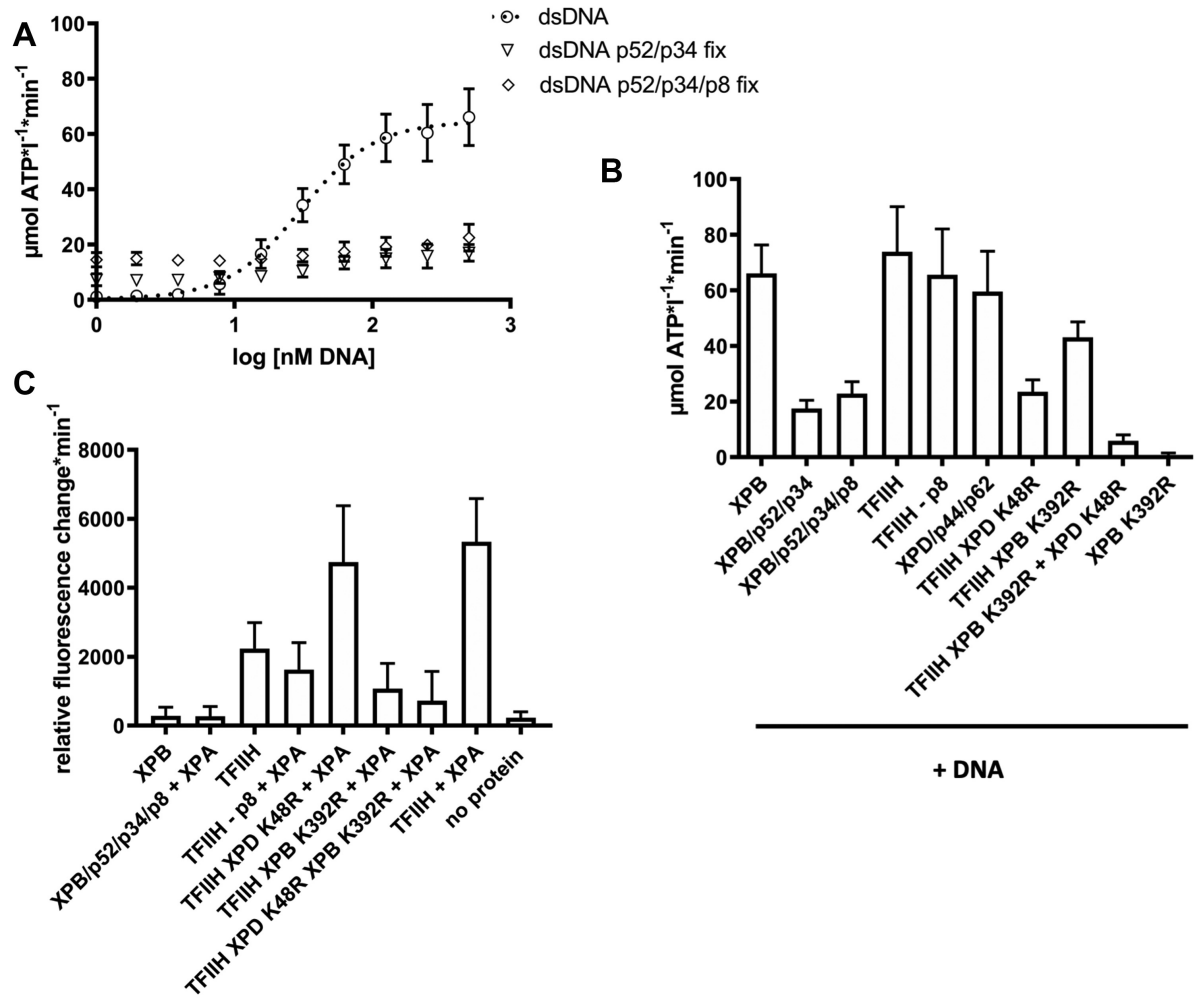
ATPase and translocase activities of ctXPB in core ctTFIIH. (**A**) ATPase activity profile of ctXPB activated by increasing amounts of dsDNA without ctp52 (open circles) and with fixed concentrations of ctp52/ctp34 (open triangles) and ctp52/ctp34/ctp8 (open rhombs). The x-axis shows the logarithmic concentration of dsDNA. The y-axis shows the NADH-consumption-derived ATP turnover rate in μmol ATP per liter per minute. *N* ≥ 6. The data for the dsDNA curve (open circles) were taken from Figure 3A for better comparison. (**B**) ATPase activity of core ctTFIIH and subunit complexes in the presence of dsDNA. The mean values and standard deviations for each condition are listed in Table 4. *N* ≥ 6. (**C**) DsDNA translocase activity of core ctTFIIH and different subunit complexes in the presence and absence of ctXPA. The mean values and standard deviations for each condition are listed in Table 4. *N* ≥ 11.

To further substantiate these observations, we investigated whether the ctp52_E359K/ctp8 complex can repress the dsDNA-mediated ctXPB activation (Figure 3D). Our data clearly show that the V_max_ of the ctXPB/ctp52_E359K/ctp8 complex is not repressed and reaches XPB/dsDNA like activity. Ctp8 by itself also does not exert any effect (Figure 3D). To assess the influence ctp8 might have on the DNA activation mechanism, we titrated dsDNA onto ctXPB/ctp52/ctp34 and ctXPB/ctp52/ctp34/ctp8 complexes and compared them to the dsDNA activation of ctXPB alone (Figure 4A). The ctXPB/ctp52/ctp34 complex is activated twofold by dsDNA from 7.1 to 17.0 μmol ATP*l^−1^*min^−1^ (Figure 4A, open triangles) and the ctXPB/ctp52/ctp34/ctp8 complex displays a similar activation from 14.3 to 24.6 μmol ATP*l^−1^*min^−1^ (Figure 4A, open rhombs). The values for ctXPB/ctp52/ctp34/ctp8 are very similar to the maximum level of activation reached by ctp52/ctp8 in the presence or absence of dsDNA (Figure 3C, 23.4 and 20.7 μmol ATP*l^−1^*min^−1^, respectively). It seems that ctp8 in this case partially compensates for the presence of dsDNA since the maximum activity value of the ctXPB/ctp52/ctp34 complex with excess of dsDNA (17.0 μmol ATP*l^−1^*min^−1^, Figure 4A open triangles) is similar to the value of the ctXPB/ctp52/ctp34/ctp8 complex (24.6 μmol ATP*l^−1^*min^−1^, Figure 4A open rhombs). It is important to note, however, that none of these complexes reached the rate of dsDNA induced activity in the sole presence of ctXPB (64.6 μmol ATP*l^−1^*min^−1^, Figure 4A, open circles).

We further investigated the ATPase activity of ctXPB in the broader context of core ctTFIIH by adding a ctXPD/ctp44/ctp62 complex to ctXPB/ctp52/ctp34/ctp8. In core ctTFIIH, the overall dsDNA induced ATPase activity is higher than for ctXPB alone with 73.8 μmol ATP*l^−1^*min^−1^ compared to 66.1 μmol ATP*l^−1^*min^−1^, respectively (Figure 4B). To delineate the influence of the two motor proteins on core ctTFIIH activity, we investigated core ctTFIIH with either the Walker A variant of XPD or XPB (ctTFIIH XPD_K48R or ctTFIIH XPB_K392R, respectively). The ctTFIIH XPD_K48R complex displayed an activity of 23.6 μmol ATP*l^−1^*min^−1^, which is almost identical to the activity of ctXPB/ctp52/ctp34 and ctXPB/ctp52/ctp34/ctp8 in the presence of dsDNA (Figure 4B and Table 4). For the ctTFIIH XPB_K392R complex we observed an activity of 43.2 μmol ATP*l^−1^*min^−1^. The single activities of both Walker A variant complexes add up to 66.8 μmol ATP*l^−1^*min^−1^ which is almost identical to our observed core ctTFIIH ATPase activity (73.8 μmol ATP*l^−1^*min^−1^). These data indicate that no further activation of the ctXPB ATPase in the core ctTFIIH environment is achieved by the presence of the additional subunits. The ctXPD/ctp44/ctp62 complex displays an ATPase activity of 59.5 μmol ATP*l^−1^*min^−1^, rendering XPD the major ATPase in core ctTFIIH. Finally, we investigated core ctTFIIH lacking ctp8. Here, we observed an activity of 65.7 μmol ATP*l^−1^*min^−1^. This corresponds to a reduction of around 8 μmol ATP*l^−1^*min^−1^ when compared to core ctTFIIH containing ctp8. This reduction in activity is in line with the observed reduction in activation of XPB’s ATPase, which emphasizes the importance of ctp8 within core ctTFIIH (43). Combined, these findings indicate that XPB’s ATPase activity is mainly regulated by p52/p8 but not by other subunits within TFIIH.

**Table 4.** Measured mean values and standard deviations (SD) from ATPase measurements of TFIIH complexes in Figure 4B

Complex	ATPase activity (μmol ATP*l^−1^*min^−1^)	SD
XPB	66.1	10.2
XPB/p52/p34	17.6	3.0
XPB/p52/p34/p8	22.9	4.4
TFIIH	73.8	16.3
TFIIH - p8	65.7	16.4
XPD/p44/p62	59.5	14.6
TFIIH K48R	23.6	4.4
TFIIH XPB K392R	43.2	5.6
TFIIH XPB K392R XPD K48R	5.9	2.1
XPB K392R	0.4	1.2

### ctXPB translocase activity is dependent on core ctTFIIH

After assessing how ctXPB’s ATPase function is regulated, we investigated how this regulation affects the translocase function of ctXPB. XPB is the essential enzymatic component of the TFIIH 5′-3′ translocase complex (25), therefore its ATPase activity should directly influence its ability to translocate on dsDNA. Since XPA was shown to stimulate TFIIH’s translocase activity (28), we also investigated the role of XPA in this process. Therefore, we used a well established triplex disruption assay to monitor ctXPB translocase activity (25,28). Reconstituted core ctTFIIH exhibits a robust translocase activity (2236 u rel. FC*min^−1^, negative control: 231 u rel. FC*min^−1^, Figure 4C and Table 5) which is further enhanced by the addition of a fourfold molar excess of ctXPA (5340 u rel. FC*min^−1^, Figure 4C and Table 5). Example time courses for the triplex displacement measurements are shown in Supplementary Figure S1B and the SDS PAGE analysis confirms the integrity and purity of the used complexes (Supplementary Figure S2). As our DNA triplex substrates would allow for more than one core ctTFIIH complex to bind, we investigated if cooperative interactions of multiple complexes on the same triplex might occur or even be necessary. We therefore performed titration experiments with either increasing amounts of ctTFIIH/ctXPA complex or increasing amounts of DNA. Both experiments indicate that translocase movement can still be observed at equimolar protein and DNA concentrations arguing against a cooperative behaviour (Supplementary Figure S6A, B). However, increasing the molar ratio of protein concentration relative to the DNA (Supplementary Figure S6A) leads to an increased activity that could be attributed to more than one core ctTFIIH being involved in translocase activity. In addition, we show that a 112 bp triplex substrate results in the same activity measured with our assay settings (500 nM ctTFIIH, 150 nM DNA) as compared to the 52 bp triplex used in this study (Supplementary Figure S6C) suggesting that the length of the DNA substrate is not a decisive factor for the translocase activity.

**Table 5.** Measured mean values and standard deviations (SD) from translocase measurements of TFIIH complexes in Figure 4C

Complex	Translocase activity (relative fluorescence change*min^−1^)	SD
XPB	283	252
XPB/p52/p34/p8 + XPA	269	288
TFIIH	2236	756
TFIIH - p8 + XPA	1628	780
TFIIH XPD K48R + XPA	4742	1642
TFIIH XPB K392R + XPA	1071	740
TFIIH XPD K48R XPB K392R + XPA	721	852
TFIIH + XPA	5340	1248
no protein	231	175

Reconstituting the core ctTFIIH/ctXPA complex with the ctXPD Walker A variant (ctXPD_K48R) has no effect on the translocase activity (4742 u rel. FC*min^−1^, Figure 4C and Table 5) whereas reconstituting the core ctTFIIH/ctXPA complex with the ctXPB Walker A variant (ctXPB_K392R) shows a strong decrease in activity (1071 u rel. FC*min^−1^, Figure 4C and Table 5). When reconstituting a core ctTFIIH/ctXPA complex containing both ctXPB and ctXPD Walker A variants, a comparable decrease in activity was observed (721 u rel. FC*min^−1^, Figure 4C and Table 5). We attribute the remaining minor activity observed for ctTFIIH/ctXPA XPB_K392R as well as for ctTFIIH/ctXPA K392R/K48R to binding events, which destabilize the triplex, rather than to a real translocase movement. These results strongly suggest that ctXPB is the major motor for translocase activity within core ctTFIIH.

Interestingly, removal of ctp8 from the core ctTFIIH/ctXPA complex also decreases the translocase activity significantly to 1628 u rel. FC*min^−1^ (Figure 4C and Table 5). To our surprise ctXPB by itself does not exhibit any measurable translocase activity within our experimental conditions (283 u rel. FC*min^−1^, Figure 4C and Table 5). Furthermore, a ctXPB/ctp52/ctp34/ctp8 complex with a 4-fold excess of ctXPA still yields no measurable translocase activity (269 u rel. FC*min^−1^).

Finally, we addressed the question if the increase in translocase activity by ctXPA is due to the stimulation of XPB’s ATPase activity. Interestingly, ctXPA does not seem to influence ctXPB’s ATPase activity in any of the analysed conditions (Supplementary Figure S7A), thus excluding the possibility that the translocase effect mediated by XPA is due to further ATPase modulation. Taken together, our data suggest that the XPB translocase function is only operating in the context of core TFIIH and the enzymatic activity is solely provided by XPB.

## DISCUSSION

Recent structural data derived from cryo-EM studies have greatly improved our understanding towards the architecture of core TFIIH and its individual components (26–28). However, little is known so far about the intricate network within core TFIIH, i.e. how the individual subunits define and regulate the activity of the entire complex. In this work we focused on the XPB helicase and investigated the regulation of XPB (i) by its direct interaction partners p52 and p8; and (ii) in the context of the entire core TFIIH, to obtain insights how the ATPase is regulated, since this function is essential for transcription and DNA repair.

Our crystal structures of ctp52 show that p52 is a flexible protein that requires its binding partners XPB and p8 to assume a complete functional state. The high structural homology of the individual p52 domains but the different orientation of the domains relative to each other within our crystal structures compared to the cryo-EM TFIIH structures provides support for this hypothesis. We cannot exclude the possibility that the domain arrangement observed in our crystal structure is a crystallization artefact due to crystal packing. The modular composition of p52, however, may well be necessary to provide the flexibility to assume different conformational states thereby fulfilling TFIIH’s functions in transcription and NER (26–28,44).

**Figure 5.**
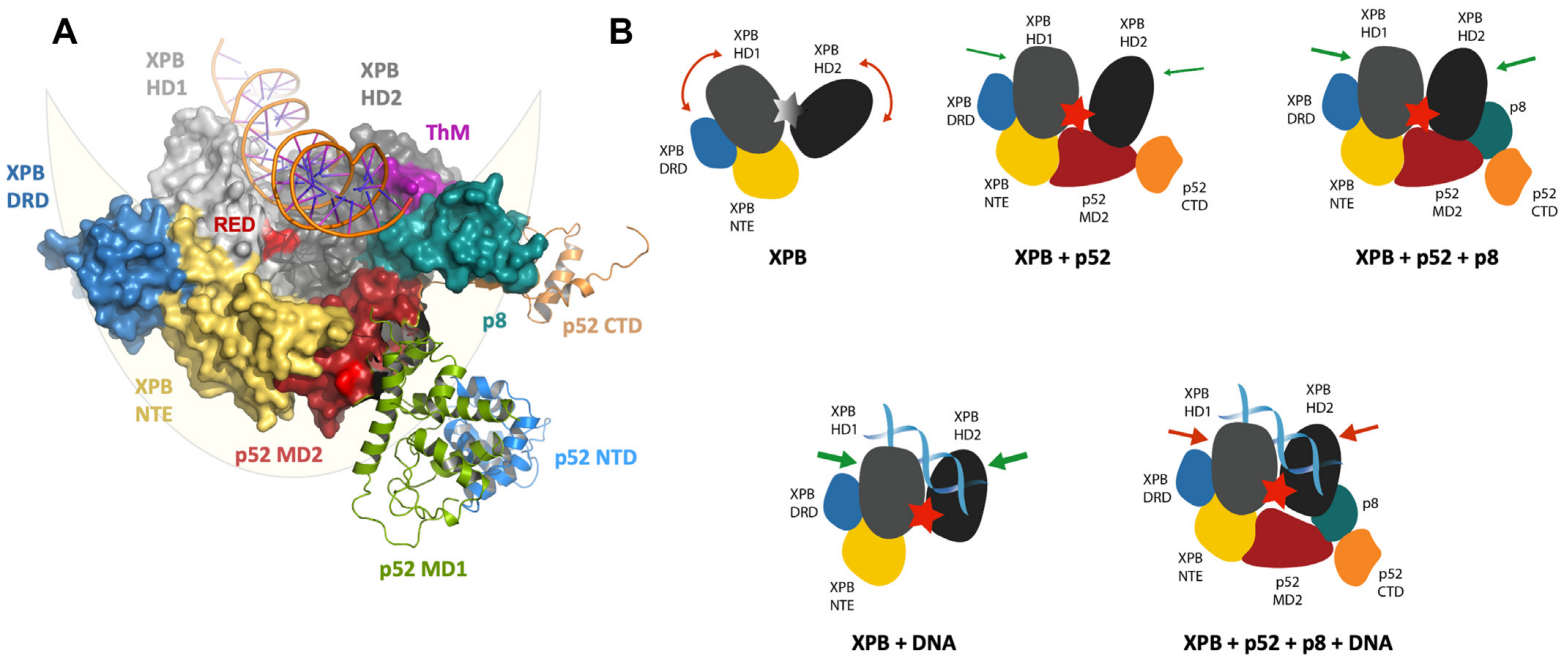
Model for p52/p8 mediated activation and restriction of XPB’s ATPase. (**A**) Structural model of the lunate-like ring that encircles the two XPB helicase domains. XPB comprises the following domains: NTE (yellow), DRD domain (blue), HD1 (light gray) and HD2 (dark gray). The ThM and RED motifs are highlighted in purple and red, respectively. P52 comprises NTD (blue), MD1 (green), MD2 (red), and CTD (orange). P8 is depicted in mint green, the DNA duplex in orange. The lunate-like ring that encircles the XPB helicase domains is schematically depicted in light yellow. Domains participating in the lunate-like ring are shown as solid surfaces. P52 domains not participating in the lunate-like ring are shown as cartoon. The structural model was derived from Schilbach, *et al.* (27) and our p52 structures. (**B**) Model of the differential XPB activation by DNA, p52/p8, p52 or p52/p8/DNA. If no activator is present, the XPB helicase domains HD1 and HD2 are too flexible and ATP hydrolysis cannot be performed. P52 binding fixes the helicase domains and thus brings them into close proximity to each other enabling ATP hydrolysis. Binding of p8 further extends the lunate-like ring and further restrains the flexibility of the two helicase domains resulting in an even higher activity. When DNA binds to XPB, the helicase domains are also restrained and brought into close proximity, leading to the highest ATPase activity. Simultaneous p52 or p52/p8 and DNA binding also results in an activation of XPB, but the activation level is limited due to the reduced flexibility of XPB.

Importantly, the high structural homology between the *C. thermophilum* and the human proteins indicates a high functional homology of the two organisms that has been described previously (11,45). In this study, we extended this system towards the analysis of XPB regulation and the functional assembly of core TFIIH. We first analysed the interaction of ctXPB and ctp52 and the effect that ctp52 exerts on ctXPB. We observed that ctp52 is sufficient to enable ctXPB’s ATPase function, which is well in line with previous work (6). To our surprise, this effect could be directly boosted by the addition of ctp8 without the presence of other core TFIIH proteins indicating a synergistic activation mechanism depending on p52/p8 only. Adding p8 to the XPB/p52 complex further enhanced the activity twofold (Figure 2A). One of the crucial players towards mediating the effect of p52 activation is the NTE of XPB. We analysed the interface formed by the NTE of XPB and MD2 of p52 through functional mutagenesis studies and showed that individual residues within this interface are critical for the p52 dependent ATPase activation. Based on the intricate network of interactions between XPB, p52, and p8 as observed in the cryo-EM structures, a sequential activation mechanism of XPB could be envisioned (Figure 5). In its apo form XPB is not able to hydrolyse ATP due to the high flexibility of the RecA like helicase domains (HD1 and HD2, Figure 5B), as observed in the crystal structures of the isolated XPB protein in which the helicase domains assume an orientation relative to each other which is not productive for ATP hydrolysis (4). Stabilisation and correct positioning is achieved by the combined action of p52 and p8. P52 interacts with the NTE and HD2 of XPB, thereby positioning HD1 and HD2 in an ATP hydrolysis competent state (26). Stability is further enhanced by p8, which also binds to HD2 of XPB. Remarkably, p8 also directly interacts with the ThM motif of XPB, clearly pointing towards a role for the proper positioning of this crucial element within XPB (Figure 1B). Combined, the interaction network comprising the MD2 domain of p52, the NTE and DRD domains of XPB and p8 leads to the formation of a lunate ring which embeds the two helicase domains of XPB, thereby permitting an ATPase compatible conformation. A recent study indicates that binding of triptolide to Cys342 of XPB might disturb the interaction with p52/p8. Cys342 is located in HD1 of XPB and points towards the cleft between the two RecA like helicase domains (Supplementary Figure S7B). Therefore, triptolide binding might push HD1 and HD2 apart from each other forcing them into an open conformation (46). This hypothesis is in agreement with our data as this enforced open conformation might counteract p52 and p8 binding via the lunate-like ring, thereby impeding enzymatic activity.

To interrogate the effect of DNA on XPB we first analysed the dsDNA-mediated activation of XPB in the absence of any other subunit and observed that the ATPase activity is about three times higher than with p52/p8, indicating that higher ATPase rates of the XPB scaffold can be achieved. To our surprise, the combination of dsDNA and p52/p8 did not lead to additional activation. On the contrary, the presence of p52/p8 limits the activation of XPB to the state that was achieved just by the addition of p52/p8 (Figure 3A) which could be attributed to limiting the *V*_max_ value of the ATPase function and not to DNA binding or changes of the apparent K_M_ for ATP (Figure 3B,C). Thus p52/p8 acts not only as a clutch (27) but also as a ’speed limiter’ ensuring that XPB does not proceed too fast either in the presence or absence of dsDNA (Figure 5B). Remarkably, this speed limitation is also not released in the presence of the other core TFIIH subunits (Figure 4B) indicating that the regulatory network formed by XPB, p52, and p8 (Figure 5A) is sufficient for the incorporation and regulation of XPB’s ATPase within the TFIIH scaffold. Disrupting the contact points between the NTE of XPB and MD2 of p52 leads to the abrogation of the control of XPB by p52/p8 and restores the activation of XPB by dsDNA (Figures 2C and 3D). It thus seems that the presence of p52/p8 on the one hand is required for XPB to assume an ATPase competent conformation but on the other hand restricts that conformation so that higher ATPase rates cannot be achieved and thereby also assumes the role as a speed limiter.

The ATPase function of XPB is a prerogative for its subsequent actions in transcription and DNA repair (reviewed in (47)) and is highly associated with its translocase activity leading to promoter opening but may also be important for NER (25,28,48). We investigated the different ATPase competent XPB complexes towards their ability to translocate on dsDNA using a triplex displacement assay. To our surprise, the ATPase function of XPB alone is not sufficient to observe translocase activity (Figure 4C). Furthermore, the complex comprising XPB/p52/p34/p8/XPA led to the same result. Since the latter complex should include all necessary elements to stimulate XPB’s translocase activity we conclude that only in a fully assembled core TFIIH translocase activity can be observed. Whether this is due to other TFIIH components aiding in productive XPB DNA binding or other TFIIH components are required for processivity remains currently unclear. However, TFIIH translocase activity is exclusively dependent on the ATPase activity of XPB, as shown by the comparison of core TFIIH assembled in the presence of the Walker A motif variants of XPD or XPB, respectively. We observed that XPA was able to boost the translocase activity about 2.4-fold which is in line with the data from Kokic *et al.* (28). XPA interacts with dsDNA above the XPB subunit of core TFIIH like a clamp, thereby further restraining XPB (28). This conformation may stimulate the translocase activity of core TFIIH. Intriguingly, this stimulation is not caused by a modulation of the ATPase activity of XPB (Supplementary Figure S7A). We thus suggest that XPA increases the processivity of the complex. Despite its size, p8 plays a highly important role with respect to the translocase activity within core TFIIH. Removal of p8 from core TFIIH causes a significant decrease in XPA stimulated translocase activity that is even lower than the activity of core TFIIH without XPA (Figure 4C). Coin *et al.* reported that p8 plays a crucial role in the recruitment of XPA (49). Hence, the impact of p8 on XPB’s translocase activity is twofold: its loss leads to a reduction in ATPase activity by affecting the overall stability of TFIIH but also impairs XPA recruitment. Combined, these observations emphasize the role of p8 as a vital factor for NER and transcription as reflected by its involvement in the disease TTD (43,50).

Our data show that XPB is a highly regulated protein. This regulation is embedded in several layers and could depend on the cellular process the protein is involved in. The maximum activation of XPB by DNA may only be important for other, less explored functions of XPB related to its localisation at the centrosome during mitosis or mRNA export (51,52) and thus requires different regulation or possibly even higher activity than in the context of TFIIH. When XPB fulfils its essential functions in transcription and NER, the main regulators are p52 and p8 that are able to activate XPB in a synergistic manner and at the same time act as a speed limiter, seemingly keeping the ATPase activity always at a constant level.

XPB’s ATPase activity is crucial for NER and transcription initiation (53), however, the requirements for XPB in both processes differ. In transcription the absence of XPB is less detrimental than its presence in an inactive state (54). Since XPB exhibits instantaneously ATPase activity when combined with p52/p8, no additional trigger is required in the TFIIH environment, rendering XPB the only functional ATPase in a non-DNA bound TFIIH, whereas XPD activity is strictly DNA-dependent (11). This DNA independent ATPase activity could be instrumental to rearrangement/recruitment processes in the initial steps of transcription. This hypothesis is supported by the low translocase activity of core TFIIH in the absence of XPA and is well in line with the function that XPB adopts in transcription, where it acts like a molecular wrench by translocating on the DNA prior to the transcription start site thereby reeling the DNA into the active site cleft of the RNAPII. This leads to torsional tension and unwinds the DNA (27) but may require only a very low translocase activity to melt several nucleotides on the DNA before RNA polymerase takes over. In NER a DNA independent ATPase activity could aid towards the initial steps of TFIIH recruitment by XPC (9) and subsequent engagement with DNA. After these initial steps, the arrival of XPA would stimulate the translocase activity to enhance the unwinding of the repair bubble necessary for XPD to engage with the DNA, yielding a repair competent complex that would also be in line with XPA’s role in lesion verification (55).

In conclusion, our analysis permitted us to decipher the tight regulatory network controlling XPB’s ATPase activity within TFIIH. We show in a stepwise approach how p52 and p8 activate XPB in an additive way. Importantly, this is independent from the activation of XPB by DNA. Despite being seen as an activator, the p52/p8 complex also limits the V_max_ of XPB’s ATPase activation by DNA and thus assumes the function of a speed limiter, which clearly dominates XPB’s regulation within TFIIH. Lastly, we show that despite its full ATPase capacity in isolation, XPB only acts as a translocase in the context of core TFIIH. Combined, our work redefines the regulatory network of the XPB ATPase and translocase shedding light on the inner regulatory mechanisms of core TFIIH in transcription and DNA repair.

## DATA AVAILABILITY

Atomic coordinates and structure factors have been deposited in the Protein Data Bank under the accession codes 6TRS (ctp52_121–514/ctp8) and 6TRU (ctp52_1–321).

## Supplementary Material

gkaa911_Supplemental_File

## References

[1] Weeda G., van Ham R.C., Masurel R., Westerveld A., Odijk H., de Wit J., Bootsma D., van der Eb A.J., Hoeijmakers J.H. Molecular cloning and biological characterization of the human excision repair gene ERCC-3. Mol. Cell. Biol. 1990; 10:2570–2581. 2111438 10.1128/mcb.10.6.2570-2581.1990 PMC360615

[2] Hwang J.R., Moncollin V., Vermeulen W., Seroz T., van Vuuren H., Hoeijmakers J.H., Egly J.-M. A 3′ → 5′ XPB helicase defect in repair/transcription factor TFIIH of xeroderma pigmentosum group B affects both DNA repair and transcription. J. Biol. Chem. 1996; 271:15898–15904. 8663148 10.1074/jbc.271.27.15898

[3] Fan L., DuPrez K.T. XPB: An unconventional SF2 DNA helicase. Prog. Biophys. Mol. Biol. 2015; 117:174–181. 25641424 10.1016/j.pbiomolbio.2014.12.005

[4] Fan L., Arvai A.S., Cooper P.K., Iwai S., Hanaoka F., Tainer J.A. Conserved XPB core structure and motifs for DNA unwinding: implications for pathway selection of transcription or excision repair. Mol. Cell. 2006; 22:27–37. 16600867 10.1016/j.molcel.2006.02.017

[5] Schaeffer L., Moncollin V., Roy R., Staub A., Mezzina M., Sarasin A., Weeda G., Hoeijmakers J.H., Egly J.-M. The ERCC2/DNA repair protein is associated with the class II BTF2/TFIIH transcription factor. EMBO J. 1994; 13:2388–2392. 8194528 10.1002/j.1460-2075.1994.tb06522.x PMC395103

[6] Coin F., Oksenych V., Egly J.-M. Distinct roles for the XPB/p52 and XPD/p44 subcomplexes of TFIIH in damaged DNA opening during nucleotide excision repair. Mol. Cell. 2007; 26:245–256. 17466626 10.1016/j.molcel.2007.03.009

[7] Compe E., Egly J.-M. TFIIH: when transcription met DNA repair. Nat. Rev. Mol. Cell Biol. 2012; 13:343–354. 22572993 10.1038/nrm3350

[8] Tirode F., Busso D., Coin F., Egly J.-M. Reconstitution of the transcription factor TFIIH: assignment of functions for the three enzymatic subunits, XPB, XPD, and cdk7. Mol. Cell. 1999; 3:87–95. 10024882 10.1016/s1097-2765(00)80177-x

[9] Oksenych V., de Jesus B.B., Zhovmer A., Egly J.-M., Coin F. Molecular insights into the recruitment of TFIIH to sites of DNA damage. EMBO J. 2009; 28:2971–2980. 19713942 10.1038/emboj.2009.230 PMC2760107

[10] Fuss J.O., Tainer J.A. XPB and XPD helicases in TFIIH orchestrate DNA duplex opening and damage verification to coordinate repair with transcription and cell cycle via CAK kinase. DNA Repair (Amst.). 2011; 10:697–713. 21571596 10.1016/j.dnarep.2011.04.028 PMC3234290

[11] Kuper J., Braun C., Elias A., Michels G., Sauer F., Schmitt D.R., Poterszman A., Egly J.-M., Kisker C. In TFIIH, XPD helicase is exclusively devoted to DNA repair. PLoS Biol. 2014; 12:e1001954. 25268380 10.1371/journal.pbio.1001954 PMC4182028

[12] Lu H., Zawel L., Fisher L., Egly J.-M., Reinberg D. Human general transcription factor IIH phosphorylates the C-terminal domain of RNA polymerase II. Nature. 1992; 358:641–645. 1495560 10.1038/358641a0

[13] Feaver W.J., Svejstrup J.Q., Henry N.L., Kornberg R.D. Relationship of CDK-activating kinase and RNA polymerase II CTD kinase TFIIH/TFIIK. Cell. 1994; 79:1103–1109. 8001136 10.1016/0092-8674(94)90040-x

[14] Roy R., Adamczewski J.P., Seroz T., Vermeulen W., Tassan J.P., Schaeffer L., Nigg E.A., Hoeijmakers J.H., Egly J.-M. The MO15 cell cycle kinase is associated with the TFIIH transcription-DNA repair factor. Cell. 1994; 79:1093–1101. 8001135 10.1016/0092-8674(94)90039-6

[15] Glover-Cutter K., Larochelle S., Erickson B., Zhang C., Shokat K., Fisher R.P., Bentley D.L. TFIIH-associated Cdk7 kinase functions in phosphorylation of C-terminal domain Ser7 residues, promoter-proximal pausing, and termination by RNA polymerase II. Mol. Cell. Biol. 2009; 29:5455–5464. 19667075 10.1128/MCB.00637-09 PMC2756882

[16] Araújo S.J., Tirode F., Coin F., Pospiech H., Syväoja J.E., Stucki M., Hübscher U., Egly J.-M., Wood R.D. Nucleotide excision repair of DNA with recombinant human proteins: definition of the minimal set of factors, active forms of TFIIH, and modulation by CAK. Genes Dev. 2000; 14:349–359. 10673506 PMC316364

[17] Coin F., Oksenych V., Mocquet V., Groh S., Blattner C., Egly J.-M. Nucleotide excision repair driven by the dissociation of CAK from TFIIH. Mol. Cell. 2008; 31:9–20. 18614043 10.1016/j.molcel.2008.04.024

[18] Peissert S., Sauer F., Grabarczyk D.B., Braun C., Sander G., Poterszman A., Egly J.-M., Kuper J., Kisker C. In TFIIH the Arch domain of XPD is mechanistically essential for transcription and DNA repair. Nat. Commun. 2020; 11:1667–1613. 32245994 10.1038/s41467-020-15241-9 PMC7125077

[19] Weeda G., van Ham R.C., Vermeulen W., Bootsma D., van der Eb A.J., Hoeijmakers J.H. A presumed DNA helicase encoded by ERCC-3 is involved in the human repair disorders xeroderma pigmentosum and Cockayne's syndrome. Cell. 1990; 62:777–791. 2167179 10.1016/0092-8674(90)90122-u

[20] Weeda G., Eveno E., Donker I., Vermeulen W., Chevallier-Lagente O., Taïeb A., Stary A., Hoeijmakers J.H., Mezzina M., Sarasin A. A mutation in the XPB/ERCC3 DNA repair transcription gene, associated with trichothiodystrophy. Am. J. Hum. Genet. 1997; 60:320–329. 9012405 PMC1712398

[21] Oh K.-S., Khan S.G., Jaspers N.G.J., Raams A., Ueda T., Lehmann A., Friedmann P.S., Emmert S., Gratchev A., Lachlan K. et al. Phenotypic heterogeneity in the XPB DNA helicase gene (ERCC3): xeroderma pigmentosum without and with Cockayne syndrome. Hum. Mutat. 2006; 27:1092–1103. 16947863 10.1002/humu.20392

[22] Bukowska B., Karwowski B.T. Actual state of knowledge in the field of diseases related with defective nucleotide excision repair. Life Sci. 2018; 195:6–18. 29305302 10.1016/j.lfs.2017.12.035

[23] Giglia-Mari G., Coin F., Ranish J.A., Hoogstraten D., Theil A., Wijgers N., Jaspers N.G.J., Raams A., Argentini M., van der Spek P.J. et al. A new, tenth subunit of TFIIH is responsible for the DNA repair syndrome trichothiodystrophy group A. Nat. Genet. 2004; 36:714–719. 15220921 10.1038/ng1387

[24] Grünberg S., Warfield L., Hahn S. Architecture of the RNA polymerase II preinitiation complex and mechanism of ATP-dependent promoter opening. Nat. Struct. Mol. Biol. 2012; 19:788–796. 22751016 10.1038/nsmb.2334 PMC3414687

[25] Fishburn J., Tomko E., Galburt E., Hahn S. Double-stranded DNA translocase activity of transcription factor TFIIH and the mechanism of RNA polymerase II open complex formation. Proc. Natl. Acad. Sci. U.S.A. 2015; 112:3961–3966. 25775526 10.1073/pnas.1417709112 PMC4386358

[26] Greber B.J., Toso D.B., Fang J., Nogales E. The complete structure of the human TFIIH core complex. Elife. 2019; 8:e44771. 30860024 10.7554/eLife.44771 PMC6422496

[27] Schilbach S., Hantsche M., Tegunov D., Dienemann C., Wigge C., Urlaub H., Cramer P. Structures of transcription pre-initiation complex with TFIIH and Mediator. Nature. 2017; 551:204–209. 29088706 10.1038/nature24282 PMC6078178

[28] Kokic G., Chernev A., Tegunov D., Dienemann C., Urlaub H., Cramer P. Structural basis of TFIIH activation for nucleotide excision repair. Nat. Commun. 2019; 10:2885. 31253769 10.1038/s41467-019-10745-5 PMC6599211

[29] Greber B.J., Nguyen T.H.D., Fang J., Afonine P.V., Adams P.D., Nogales E. The cryo-electron microscopy structure of human transcription factor IIH. Nature. 2017; 549:414–417. 28902838 10.1038/nature23903 PMC5844561

[30] Li M.Z., Elledge S.J. Harnessing homologous recombination in vitro to generate recombinant DNA via SLIC. Nat. Methods. 2007; 4:251–256. 17293868 10.1038/nmeth1010

[31] Wang W., Malcolm B.A. Two-stage PCR protocol allowing introduction of multiple mutations, deletions and insertions using QuikChange Site-Directed Mutagenesis. BioTechniques. 1999; 26:680–682. 10343905 10.2144/99264st03

[32] Walden H. Selenium incorporation using recombinant techniques. Acta Crystallogr. D. Biol. Crystallogr. 2010; 66:352–357. 20382987 10.1107/S0907444909038207 PMC2852298

[33] Kabsch W. XDS. Acta Crystallogr. D. Biol. Crystallogr. 2010; 66:125–132. 20124692 10.1107/S0907444909047337 PMC2815665

[34] Tickle I., Flensburg C., Keller P., Paciorek W., Sharff A., Vonrhein C., Bricogne G. STARANISO. 2020; http://staraniso.globalphasing.org/cgi-bin/staraniso.cgi.

[35] Vonrhein C., Blanc E., Roversi P., Bricogne G. Automated structure solution with autoSHARP. Methods Mol. Biol. 2007; 364:215–230. 17172768 10.1385/1-59745-266-1:215

[36] McCoy A.J., Grosse-Kunstleve R.W., Adams P.D., Winn M.D., Storoni L.C., Read R.J. Phaser crystallographic software. J. Appl. Crystallogr. 2007; 40:658–674. 19461840 10.1107/S0021889807021206 PMC2483472

[37] Emsley P., Lohkamp B., Scott W.G., Cowtan K. Features and development of Coot. Acta Crystallogr. D. Biol. Crystallogr. 2010; 66:486–501. 20383002 10.1107/S0907444910007493 PMC2852313

[38] Murshudov G.N., Skubák P., Lebedev A.A., Pannu N.S., Steiner R.A., Nicholls R.A., Winn M.D., Long F., Vagin A.A. REFMAC5 for the refinement of macromolecular crystal structures. Acta Crystallogr. D. Biol. Crystallogr. 2011; 67:355–367. 21460454 10.1107/S0907444911001314 PMC3069751

[39] Bricogne G., Blanc E., Brandl M., Flensburg C., Keller P., Paciorek W., Pietro Roversi, Sharff A., Smart O., Vonrhein C. et al. Buster. 2017;

[40] Kainov D., Vitorino M., Cavarelli J., Poterszman A., Egly J.-M. Structural basis for group A trichothiodystrophy. Nat. Struct. Mol. Biol. 2008; 15:980–984. 19172752 10.1038/nsmb.1478

[41] Fregoso M., Lainé J.-P., Aguilar-Fuentes J., Mocquet V., Reynaud E., Coin F., Egly J.-M., Zurita M. DNA repair and transcriptional deficiencies caused by mutations in the Drosophila p52 subunit of TFIIH generate developmental defects and chromosome fragility. Mol. Cell. Biol. 2007; 27:3640–3650. 17339330 10.1128/MCB.00030-07 PMC1899989

[42] Smurnyy Y., Cai M., Wu H., McWhinnie E., Tallarico J.A., Yang Y., Feng Y. DNA sequencing and CRISPR-Cas9 gene editing for target validation in mammalian cells. Nat. Chem. Biol. 2014; 10:623–625. 24929529 10.1038/nchembio.1550

[43] Theil A.F., Hoeijmakers J.H.J., Vermeulen W. TTDA: big impact of a small protein. Exp. Cell Res. 2014; 329:61–68. 25016283 10.1016/j.yexcr.2014.07.008

[44] Yan C., Dodd T., He Y., Tainer J.A., Tsutakawa S.E., Ivanov I. Transcription preinitiation complex structure and dynamics provide insight into genetic diseases. Nat. Struct. Mol. Biol. 2019; 26:397–406. 31110295 10.1038/s41594-019-0220-3 PMC6642811

[45] Radu L., Schoenwetter E., Braun C., Marcoux J., Koelmel W., Schmitt D.R., Kuper J., Cianférani S., Egly J.M., Poterszman A. et al. The intricate network between the p34 and p44 subunits is central to the activity of the transcription/DNA repair factor TFIIH. Nucleic Acids Res. 2017; 45:10872–10883. 28977422 10.1093/nar/gkx743 PMC5737387

[46] Uriostegui-Arcos M., Aguayo-Ortiz R., Valencia-Morales M.D.P., Melchy-Pérez E., Rosenstein Y., Dominguez L., Zurita M. Disruption of TFIIH activities generates a stress gene expression response and reveals possible new targets against cancer. Open Biol. 2020; 10:200050. 32543350 10.1098/rsob.200050 PMC7333893

[47] Compe E., Egly J.-M. Nucleotide excision repair and transcriptional regulation: TFIIH and beyond. Annu. Rev. Biochem. 2016; 85:265–290. 27294439 10.1146/annurev-biochem-060815-014857

[48] Tomko E.J., Fishburn J., Hahn S., Galburt E.A. TFIIH generates a six-base-pair open complex during RNAP II transcription initiation and start-site scanning. Nat. Struct. Mol. Biol. 2017; 24:1139–1145. 29106413 10.1038/nsmb.3500 PMC5741190

[49] Coin F., Proietti De Santis L., Nardo T., Zlobinskaya O., Stefanini M., Egly J.-M. p8/TTD-A as a repair-specific TFIIH subunit. Mol. Cell. 2006; 21:215–226. 16427011 10.1016/j.molcel.2005.10.024

[50] Theil A.F., Nonnekens J., Steurer B., Mari P.-O., de Wit J., Lemaitre C., Marteijn J.A., Raams A., Maas A., Vermeij M. et al. Disruption of TTDA results in complete nucleotide excision repair deficiency and embryonic lethality. PLos Genet. 2013; 9:e1003431. 23637614 10.1371/journal.pgen.1003431 PMC3630102

[51] Weber A., Chung H.-J., Springer E., Heitzmann D., Warth R. The TFIIH subunit p89 (XPB) localizes to the centrosome during mitosis. Cell. Oncol. 2010; 32:121–130. 20208140 10.3233/CLO-2009-0509 PMC4619175

[52] Mizuki F., Namiki T., Sato H., Furukawa H., Matsusaka T., Ohshima Y., Ishibashi R., Andoh T., Tani T. Participation of XPB/Ptr8p, a component of TFIIH, in nucleocytoplasmic transport of mRNA in fission yeast. Genes Cells. 2007; 12:35–47. 17212653 10.1111/j.1365-2443.2006.01032.x

[53] Egly J.-M., Coin F. A history of TFIIH: two decades of molecular biology on a pivotal transcription/repair factor. DNA Repair (Amst.). 2011; 10:714–721. 21592869 10.1016/j.dnarep.2011.04.021

[54] Sandoz J., Coin F. Unified promoter opening steps in eukaryotic gene expression. Oncotarget. 2017; 8:84614–84615. 29156658 10.18632/oncotarget.21387 PMC5689548

[55] Li C.-L., Golebiowski F.M., Onishi Y., Samara N.L., Sugasawa K., Yang W. Tripartite DNA lesion recognition and verification by XPC, TFIIH, and XPA in nucleotide excision repair. Mol. Cell. 2015; 59:1025–1034. 26384665 10.1016/j.molcel.2015.08.012 PMC4617536

