# Supplementary material for: How to limit the speed of a motor: the intricate regulation of the XPB ATPase and translocase in TFIIH
Source: Nucleic Acids Res. 2020 Nov 16;48(21):12282–96. doi: 10.1093/nar/gkaa911 (PMC7708078; doi:10.1093/nar/gkaa911)
Supplement: gkaa911_Supplemental_File [file gkaa911_supplemental_file.docx]

**Supplementary Information:**


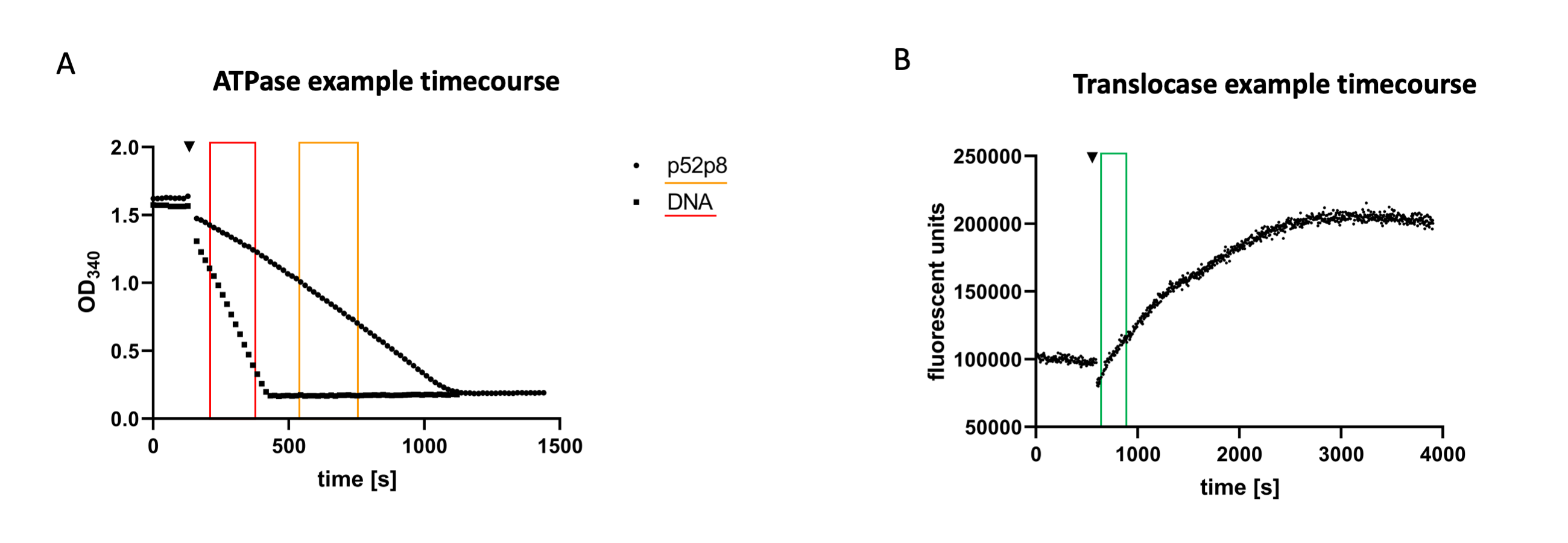


**Supplementary Figure 1: Example timecourses for ATPase and triplex displacement assays. (A)** Raw data for the ATPase assay with ctXPB activated by ctp52/ctp8 (circles) or dsDNA (squares). The arrow indicates the time point of injection. The ranges selected for the kinetic calculations are shown in red (dsDNA) and orange (ctp52/ctp8). **(B)** Raw data for triplex displacement assay with core ctTFIIH + ctXPA. The arrow indicates the time point of injection. The range selected for the kinetic calculation is shown in green. The ranges were selected to cover initial linear velocity.


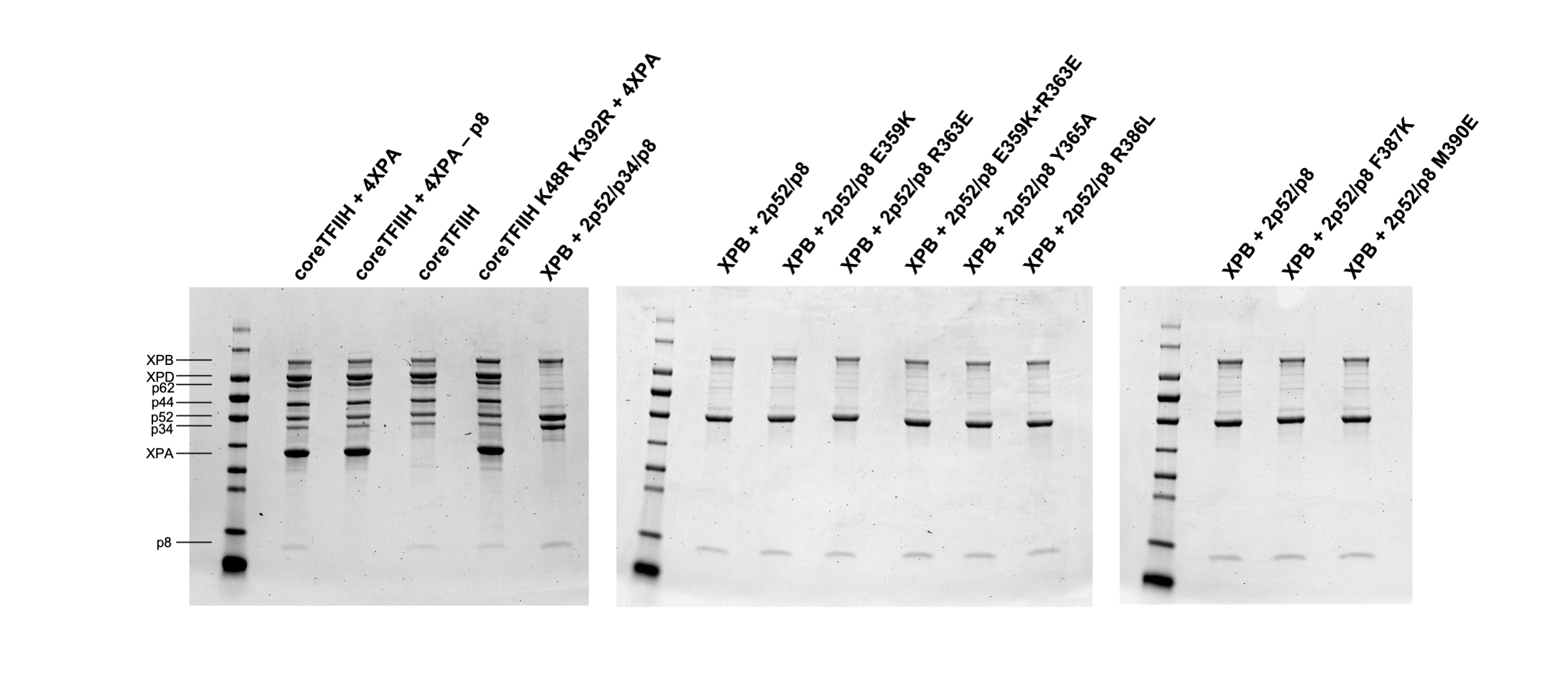


**Supplementary Figure 2: SDS PAGE analyses of the complexes used for ATPase and triplex displacement assays.** The various complexes used in this study were loaded on a 4-20 % Mini-PROTEAN TGX Gel (Bio-Rad) and stained with Coomassie Brilliant Blue G250 (Carl Roth).


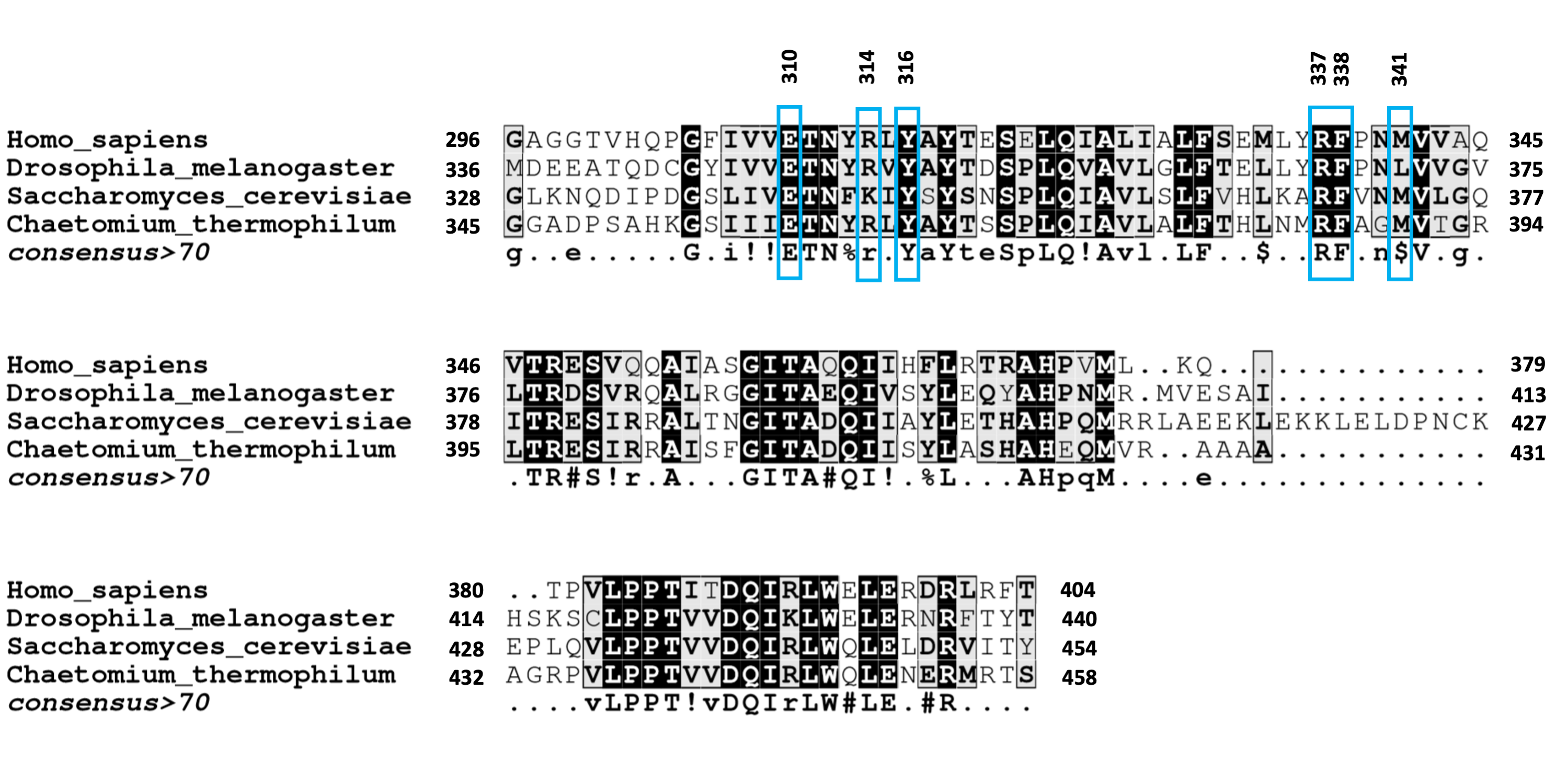


**Supplementary Figure 3: Multiple sequence alignment of p52 MD2.** Protein sequences of p52 MD2 from *Homo sapiens*, *Chaetomium thermophilum*, *Saccharomyces cerevisiae* and *Drosophila melanogaster* were aligned using T-coffee {DiTommaso:2011kx} and ESPript 3.0 {Robert:2014fy}. The p52 residues mutated in this study are highlighted in blue. The numbering of these residues corresponds to the human sequence.


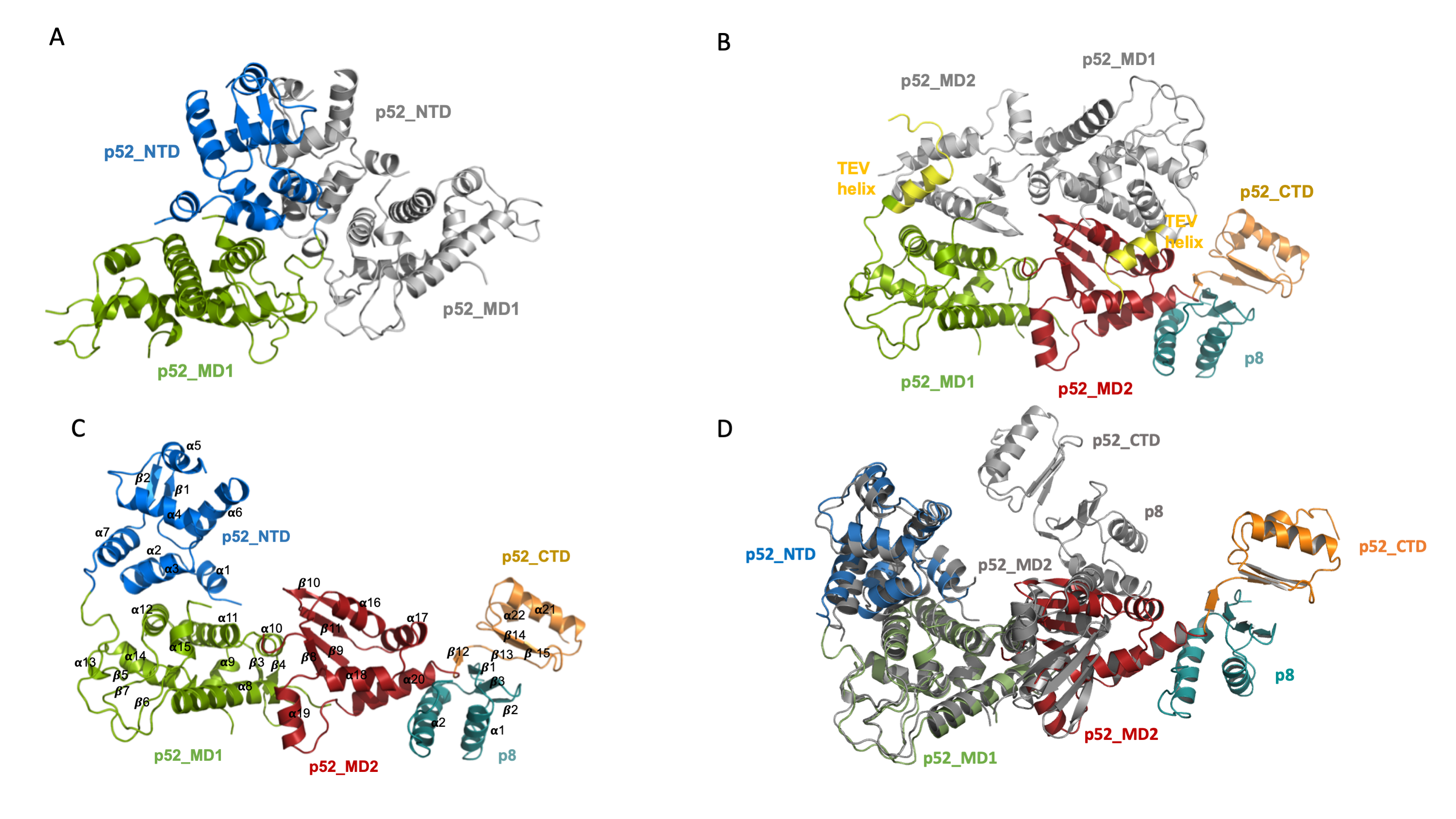


**Supplementary Figure 4: Crystal structure of full length ctp52 in complex with ctp8.** **(A)** Crystal structure of the ctp52_1-321 construct that comprises the NTD and MD1 of ctp52. There are four molecules in the asymmetric unit. Two of them form a dimer. The second molecule of the dimer is shown in grey. **(B)** Crystal structure of the ctp52_121-514 construct that comprises MD1, MD2 and CTD of ctp52, as well as ctp8. The TEV helix is involved in dimerization. There are two molecules in the asymmetric unit, the second molecule is shown in grey. **(C)** Superposition of the two crystal structures from **(A)** and **(B)** via MD1 generates the full length ctp52 model. **(D)** Superposition of our ctp52 structure (colored) with the human p52 and p8 structure (grey) in the context of TFIIH {Greber:2019dq}.


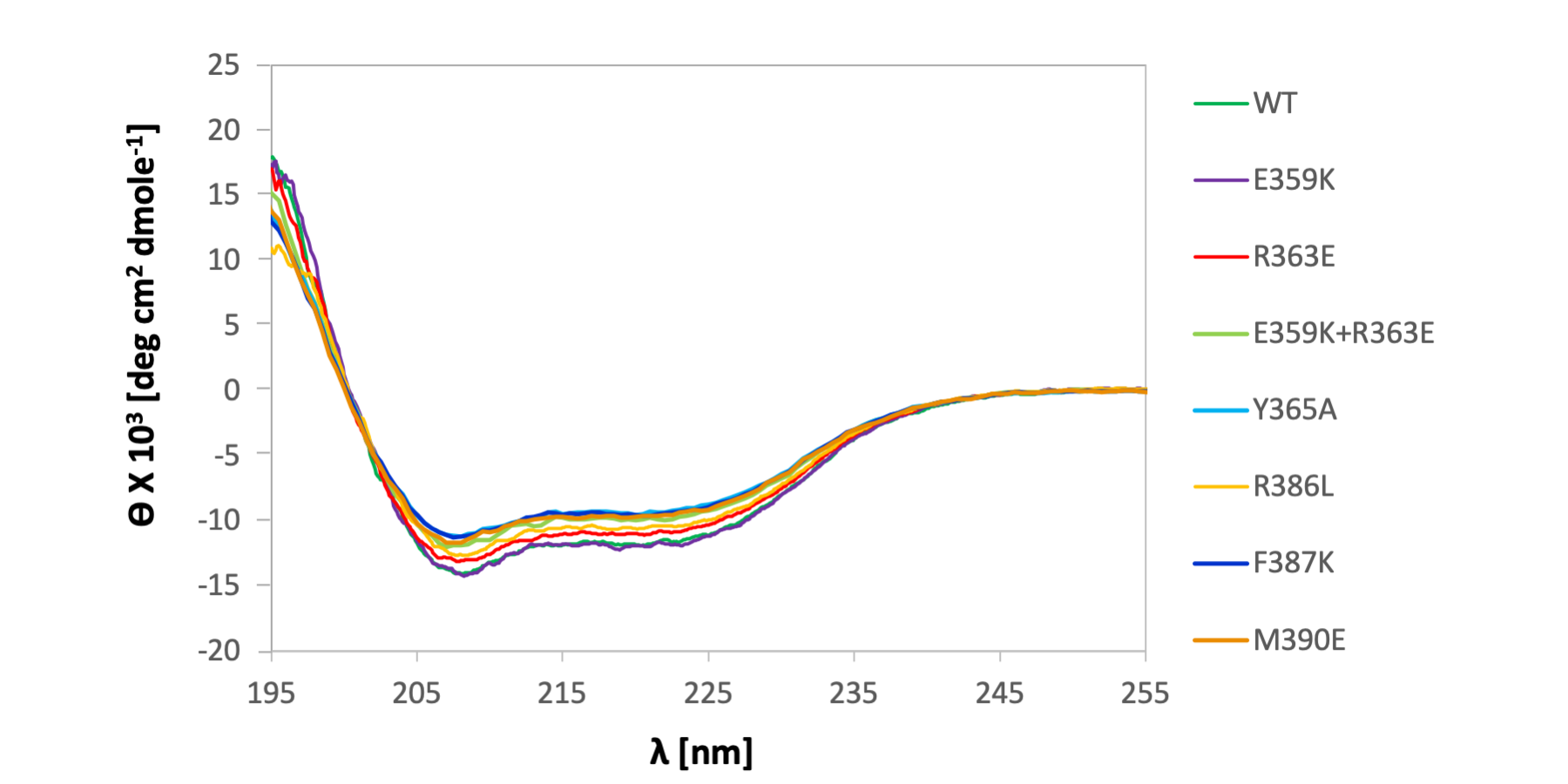


**Supplementary Figure 5: CD spectroscopy of ctp52/ctp8 wild type and variants.** Analysis of ctp52 wild type and variants in complex with ctp8 via CD spectroscopy.


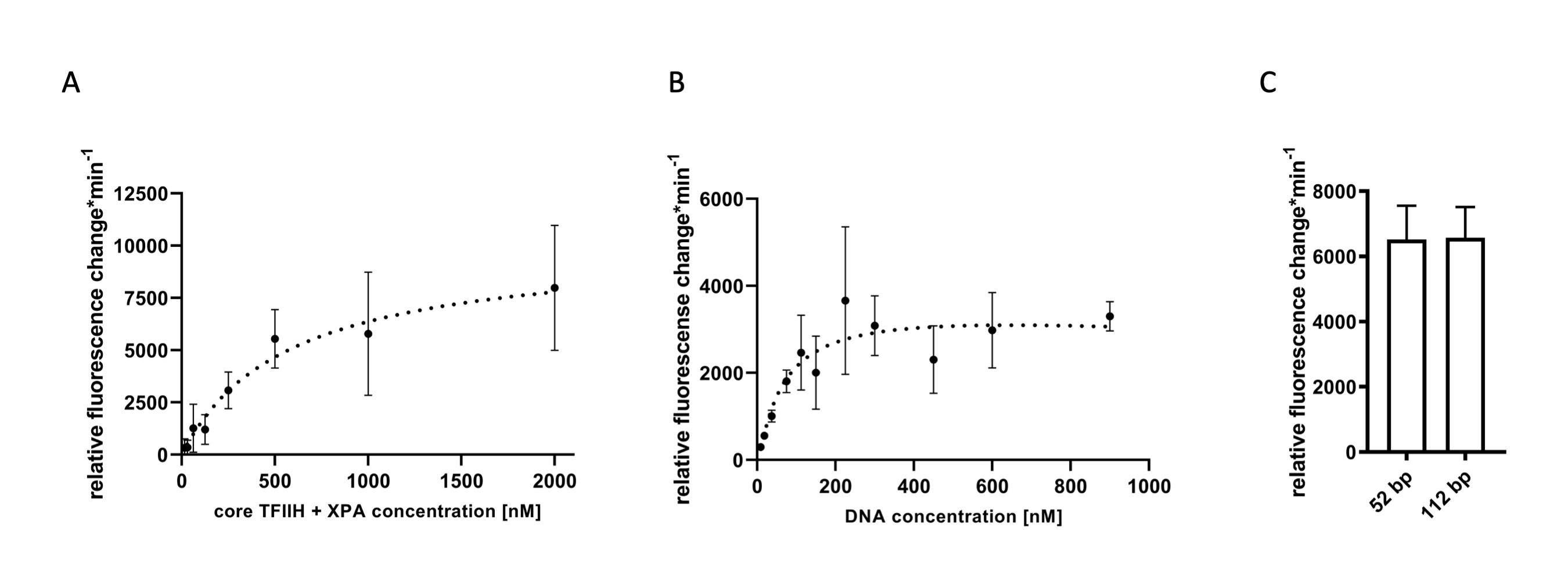


**Supplementary Figure 6: Translocase titration experiments.** **(A)** Core ctTFIIH + ctXPA titration with a fixed triplex DNA concentration. N = 5. **(B)** Triplex DNA titration with a fixed core ctTFIIH + ctXPA concentration. For this measurement, the gain was reduced to 1700 and the slope of the fluorescence increase was subtracted from the baseline prior to ATP titration. N = 4. **(C)** Core ctTFIIH + ctXPA with different triplex DNA lengths. N ≥ 7.


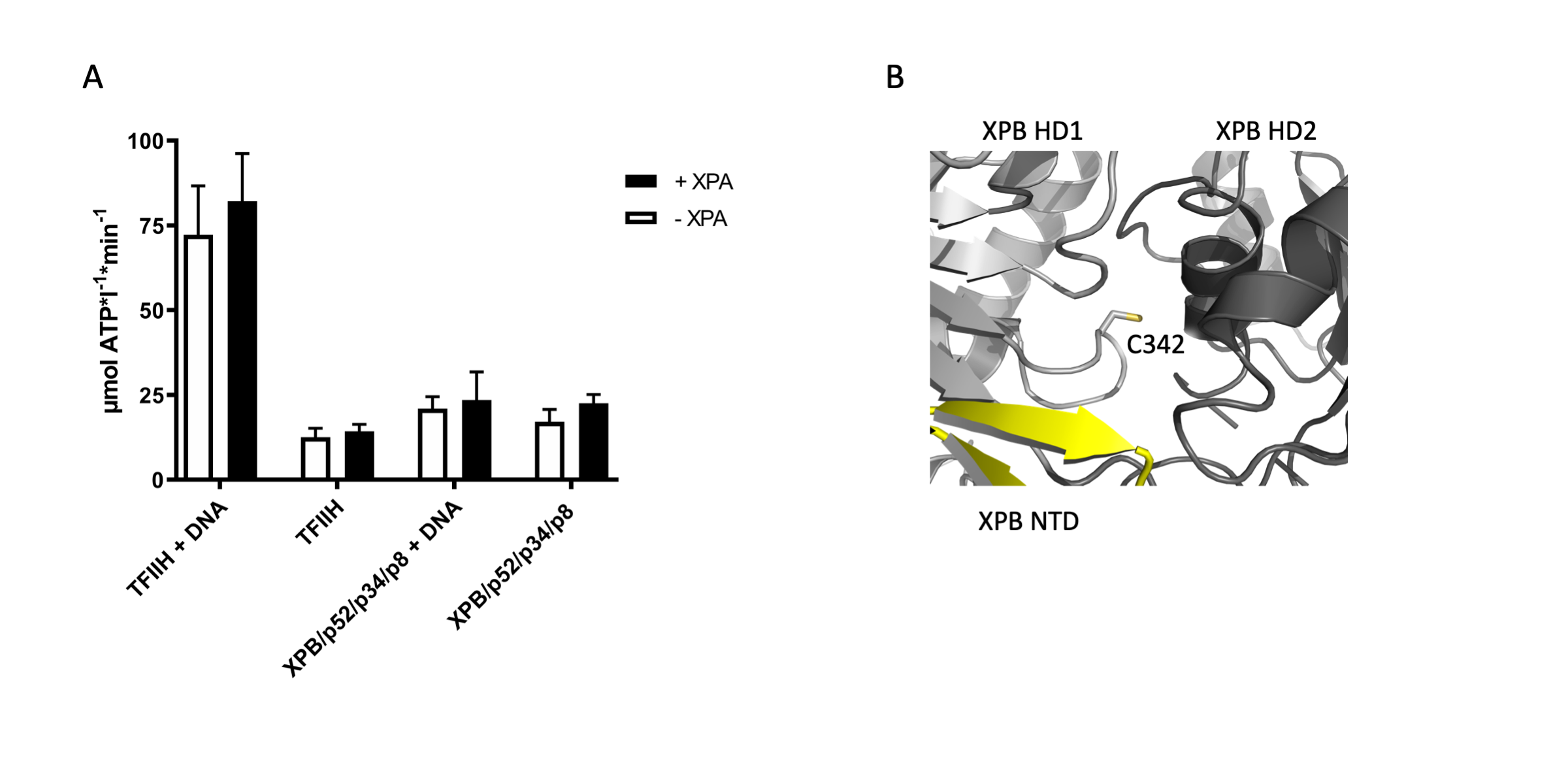


**Supplementary Figure 7: ATPase activity of different ctTFIIH subunit complexes in the presence and absence of ctXPA and DNA.** **(A)** Different core ctTFIIH complexes with or without ctXPA were analysed in the presence or absence of dsDNA. The y-axis shows the NADH-consumption-derived ATP turnover rate in µmol ATP per liter per minute. N ≥ 5. **(B)** Model of human XPB showing the Triptolide binding site at C342. Structural information taken from 6RO4.
